# Hinged and Wide: A New P^P Ligand for Emissive [Cu(P^P)(N^N)][PF_6_] Complexes

**DOI:** 10.3390/molecules24213934

**Published:** 2019-10-31

**Authors:** Sarah Keller, Matthias Bantle, Alessandro Prescimone, Edwin C. Constable, Catherine E. Housecroft

**Affiliations:** 1 Department of Chemistry, University of Basel, BPR 1096, Mattenstrasse 24a, CH-4058 Basel, Switzerland; sarah.keller@chimieparistech.psl.eu (S.K.); mattu.bantle@gmail.com (M.B.); alessandro.prescimone@unibas.ch (A.P.); edwin.constable@unibas.ch (E.C.C.); 2 Chimie ParisTech, PSL University CNRS, Institute of Chemistry for Life and Health Sciences, Laboratory for Inorganic Chemical Biology, 11 rue Pierre et Marie Curie, F-75005 Paris, France

**Keywords:** copper(I), bis(phosphane), 2,2′-bipyridine, X-ray structure, photophysics, TADF

## Abstract

Heteroleptic [Cu(BIPHEP)(N^N)][PF_6_] complexes (BIPHEP = 1,1′-biphenyl-2,2′-diylbis(diphenylphosphane)), in which N^N is 2,2′-bipyridine (bpy), 6-methyl-2,2′-bipyridine (6-Mebpy), 6-ethyl-2,2′-bipyridine (6-Etbpy), or 5,5′-dimethyl-2,2′-bipyridine (5,5′-Me_2_bpy), have been synthesized and characterized using multinuclear NMR spectroscopies and electrospray ionization mass spectrometry. The single crystal structures of [Cu(BIPHEP)(bpy)][PF_6_]∙CH_2_Cl_2_, [Cu(BIPHEP)(5,5′-Me_2_bpy)][PF_6_]∙CH_2_Cl_2_, [Cu(BIPHEP)(6-Mebpy)][PF_6_]∙Et_2_O∙0.5H_2_O and [Cu(BIPHEP)(6-Etbpy)][PF_6_] confirm distorted tetrahedral {Cu(P^P)(N^N)} coordination environments. Each compound shows a quasi-reversible Cu^+^/Cu^2+^ process. In deaerated solution, the compounds are weak emitters. Powdered samples are yellow emitters (*λ*_em_^max^ in the range 558–583 nm) and [Cu(BIPHEP)(5,5′-Me_2_bpy)][PF_6_] exhibits the highest photoluminescence quantum yield (PLQY = 14%). On cooling to 77 K (frozen 2-methyloxolane), the emission maxima are red-shifted and the excited state lifetimes increase from *τ*_1/2_ < 8 μs, to *τ*_1/2_ values of up to 53 μs, consistent with the compounds with N^N = 6-Mebpy, 6-Etbpy and 5,5′-Me_2_bpy exhibiting thermally activated delayed fluorescence (TADF).

## 1. Introduction

Organic light-emitting diodes (OLEDs) and light-emitting electrochemical cells (LECs) contribute to modern solid-state lighting technologies, and significant advances in the performances of these devices have been made in recent years [1,2,3,4,5,6]. The discovery of the photophysical phenomenon of thermally activated delayed fluorescence (TADF), which allows both singlet and triplet excitons to be harvested for light emission, has injected huge excitement into the field [7,8,9]. In the TADF process, after excitation of a compound into the lowest-lying singlet excited state, fast intersystem crossing to the excited triplet state, at a slightly lower energy (<0.38 eV [9]), takes place. As the triplet state is long lived, and emission as phosphorescence is rather slow, the thermal energy at room temperature allows repopulation of the singlet excited state from which fluorescence occurs. The emission at room temperature is, therefore, a combination of phosphorescence from the triplet excited state and fluorescence from the singlet excited state, allowing, in theory, a 100% internal quantum efficiency. Although organic compounds have been widely used for TADF applications [10,11] metal complexes are particularly effective and can be easily tuned through ligand modification to possess desired excited state energies [9]. The development of LECs which contain ionic copper(I) complexes in the emissive layer is especially appealing, since it capitalizes on the use of an Earth-abundant metal and [Cu(P^P)(N^N)]^+^ complexes (N^N = diimine, P^P = bisphosphane) which exhibit TADF [9]. Application of these copper(I) coordination compounds follows early results from McMillin and co-workers, who were the first to report that copper(I) complexes comprising PPh_3_ or P^P ligands and 2,2′-bipyridine (bpy) or 1,10-phenanthroline (phen) exhibited low-lying metal-to-ligand charge transfer (MLCT) excited states [12,13]. 

**Scheme 1:**
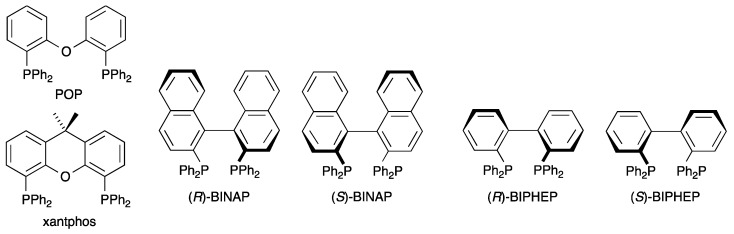
Structures of the bis(phosphane) ligands POP, xantphos, BINAP and BIPHEP.

To date, the P^P ligands most thoroughly explored for incorporation into emissive [Cu(P^P)(N^N)]^+^ complexes have been 4,5-bis(diphenylphosphanyl)-9,9-dimethyl-9*H*-xanthene (xantphos, IUPAC PIN (9,9-dimethyl-9*H*-xanthene-4,5-diyl)bis(diphenylphosphane)) and bis(2-(diphenylphosphanyl)phenyl)ether (POP, IUPAC PIN oxydi(2,1-phenylene)]bis(diphenylphosphane)) (Scheme 1). Both are classed as wide bite–angle bisphosphanes [14], and the P–Cu–P bite angles in the solid-state structures of [Cu(POP)(bpy)][PF_6_]**^.^**CHCl_3_, [Cu(POP)(bpy)][PF_6_]**^.^**Et_2_O and [Cu(xantphos)(bpy)][PF_6_] are 115.00(3)° [15], 111.97(3)° [16] and 113.816(14) [17], respectively. A wide variety of bpy-based ligands have been combined with POP and xantphos in systematic investigations of [Cu(P^P)(N^N)]^+^ emitters [3,17,18,19,20,21,22,23] and references cited therein]. In contrast, variations of the P^P ligand (other than POP and xantphos) have received less attention. Replacing the PPh_2_ units in xantphos with PMes_2_ units (Mes = mesityl) leads to a P–Cu–P angle in [Cu(xantphosMes_2_)(bpy)][PF_6_] (xantphosMes_2_ = 9,9-dimethyl-9*H*-xanthene-4,5-diyl)bis[di(2,4,6-trimethylphenyl)phosphane]) of 115.96(2)° [24]. We have observed that introducing P^t^Bu_2_ in place of PPh_2_ groups in the xantphos derivative 9,9-dimethyl-9*H*-xanthene-4,5-diyl)bis[di(*tert*-butyl)phosphane] is detrimental, and gives rise to very weakly emitting [Cu(P^P)(N^N)][PF_6_] compounds, likely due to the vibrational quenching effects of the *tert*-butyl substituents [25]. Retaining aromatic substituents is therefore critical for efficient energy to light conversion. Two sterically demanding ligands that fulfil this requirement are 1,1′-biphenyl-2,2′-diylbis(diphenylphosphane) (BIPHEP) and 1,1′-binaphthalene-2,2′-diylbis(diphenylphosphane) (BINAP) (Scheme 1). A search of the Cambridge Structural Database (v. 5.40 with February 2019 updates [26]) for {Cu(BINAP)(N^N)}-containing compounds, in which N^N is constrained to a chelating ligand, reveals only six entries [27,28,29,30] and for these, the range of P–Cu–P bond angles is 99.25–105.13°. Both BINAP and BIPHEP are expected to exhibit atropisomerism, but, at room temperature, interconversion of (*R*)- and (*S*)-BINAP is slow, whereas the atropisomers (*R*)- and (*S*)-BIPHEP (Scheme 1) interconvert rapidly in solution [31]. Coordination of BIPHEP to a metal centre can lead to chiral metal coordination compounds. The preparation and photophysical properties of [Cu(BINAP)(N^N)]^+^ complexes have been reported [28], but, even though BIPHEP is commercially available, no [Cu(BIPHEP)(N^N)]^+^ complexes appear to have been described. We were, therefore, inspired to investigate the structural and solution properties of such complexes. In this work, we describe the preparation and characterization of [Cu(BIPHEP)(bpy)][PF_6_], along with three derivatives, in which the bpy metal-binding domain bears alkyl substituents in the 5- or 6-positions.

## 2. Results and Discussion

### 2.1. Synthesis and Characterization of [Cu(BIPHEP)(N^N][PF_6_] Complexes

The [Cu(BIPHEP)(N^N)][PF_6_] complexes, in which N^N is bpy, 6-Mebpy, 6-Etbpy or 5,5′-Me_2_bpy, were prepared by adding CH_2_Cl_2_ solutions containing both BIPHEP and the respective 2,2′-bipyridine ligand to CH_2_Cl_2_ solutions of [Cu(MeCN)_4_][PF_6_]. This led to an immediate colour change from colourless to yellow, indicating the formation of the heteroleptic complexes without ligand redistribution; formation of a homoleptic [Cu(N^N)_2_][PF_6_] complex would have been accompanied by the development of a distinctive red colour. Attempts to prepare the analogous complex [Cu(BIPHEP)(6,6′-Me_2_bpy)][PF_6_] resulted in a mixture of homoleptic complexes as well as the desired heteroleptic compound, and since equilibration between hetero- and homoleptic complexes persisted in the solution, this reaction was not investigated further. The compounds [Cu(BIPHEP)(bpy)][PF_6_], [Cu(BIPHEP)(6-Mebpy)][PF_6_], [Cu(BIPHEP)(6-Etbpy)][PF_6_] and [Cu(BIPHEP)(5,5′-Me_2_bpy)][PF_6_] were characterized by elemental analysis, mass spectrometry, and multinuclear NMR spectroscopies. 

**Scheme 2:**
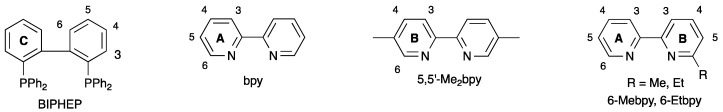
The structure of the ligands and numbering scheme for NMR spectroscopic assignments. The phenyl rings in BIPHEP are labelled **D**.

**Figure 1:**
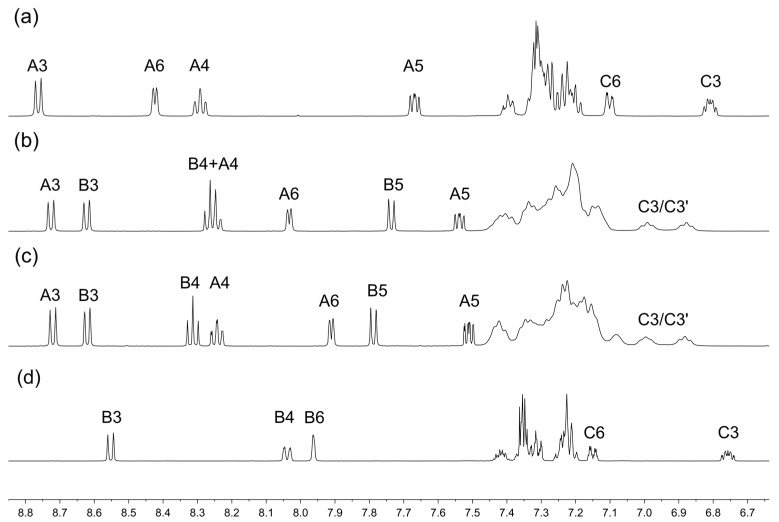
^1^H-NMR spectra (aromatic regions, 500 MHz, acetone-*d*_6_, 298 K) of (**a**) [Cu(BIPHEP)(bpy)][PF_6_], (**b**) [Cu(BIPHEP)(6-Mebpy)][PF_6_], (**c**) [Cu(BIPHEP)(6-Etbpy)][PF_6_] and (**d**) [Cu(BIPHEP)(5,5′-Me_2_bpy)][PF_6_].

**Figure 2:**
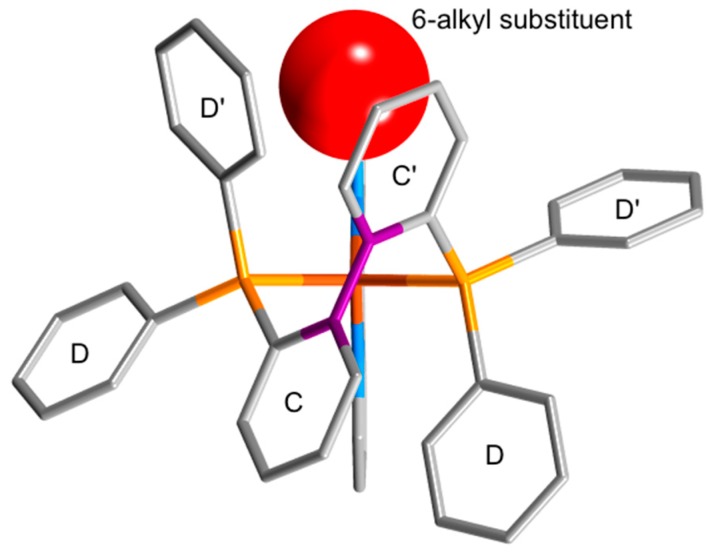
Modelled structure (not energy minimized) of [Cu(BIPHEP)(6-Rbpy)]^+^, in which the alkyl group R is represented by the red sphere and the trans-annular bond in the biphenyl unit is shown in purple. Partial rotation about the latter renders the two D’ rings equivalent and the two D rings equivalent, but the pair C and C’, and the pair D and D’, remain non-equivalent.

**Figure 3:**
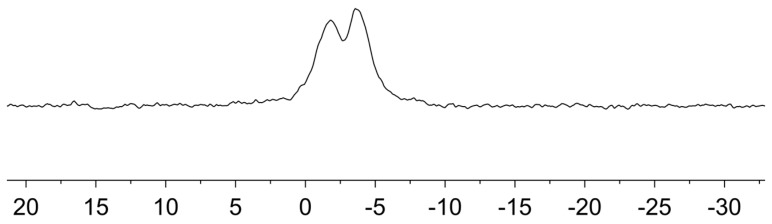
The signal arising from the [Cu(BIPHEP)(6-Mebpy)]^+^ cation in the ^31^P{^1^H} NMR spectrum (202 MHz, acetone-*d*_6_, 298 K) of [Cu(BIPHEP)(6-Mebpy)][PF_6_].

In the ESI-MS spectrum of each compound, the base peak was assigned to the [M–PF_6_]^+^ ion (Figures S1–S4, see Supporting Information). The room temperature ^1^H and ^13^C{^1^H} NMR spectra were recorded in acetone-*d*_6_ solution and signals were assigned (see Materials and Methods section) using 2D-methods. Aromatic ring and atom labelling are shown in Scheme 2. COSY, HMQC and HMBC spectra are displayed in Figures S5–S16 (see Supporting Information). Figure 1 shows that the signals arising from the coordinated bpy, 6-Mebpy, 6-Etbpy and 5,5′-Me_2_bpy ligands are well resolved and sharp. The non-equivalent pyridine rings in [Cu(BIPHEP)(6-Mebpy)][PF_6_] and [Cu(BIPHEP)(6-Etbpy)][PF_6_] (Figure 1b,c) were readily distinguished, starting with the characteristic signal for proton H^A6^ in the COSY spectrum and using the NOESY signal between the 6-alkyl group and proton H^B5^. In [Cu(BIPHEP)(bpy)][PF_6_] and [Cu(BIPHEP)(5,5′-Me_2_bpy)][PF_6_], the ^1^H and ^13^C{^1^H} NMR spectra at room temperature reveal that the rings (rings C in Scheme 1) in the biphenyl unit of BIPHEP are equivalent. Consistent with this is the observation of a single signal in the ^31^P{^1^H} NMR spectrum for the cation, at *δ* −1.2 ppm and *δ* −0.6 ppm, respectively, for [Cu(BIPHEP)(bpy)][PF_6_] and [Cu(BIPHEP)(5,5′-Me_2_bpy)][PF_6_]. In [Cu(BIPHEP)(6-Mebpy)][PF_6_] and [Cu(BIPHEP)(6-Etbpy)][PF_6_], the rings of the biphenyl unit are non-equivalent, and this is illustrated by comparing Figure 1a,d with Figure 1b,c, with a splitting of the signal for the H^C3^ protons. This is consistent with the modelled structure [32] shown in Figure 2; the biphenyl rings remain non-equivalent, even with partial rotation about the trans-annular C–C bond of the biphenyl unit. At 298 K, the solution ^31^P{^1^H} NMR spectrum of [Cu(BIPHEP)(6-Mebpy)][PF_6_] (Figure 3) and [Cu(BIPHEP)(6-Etbpy)][PF_6_] (Figure S17 in the Supporting Information) exhibits two signals of similar chemical shifts, in addition to the signal for the [PF_6_]^−^ ion. 

**Figure 4:**
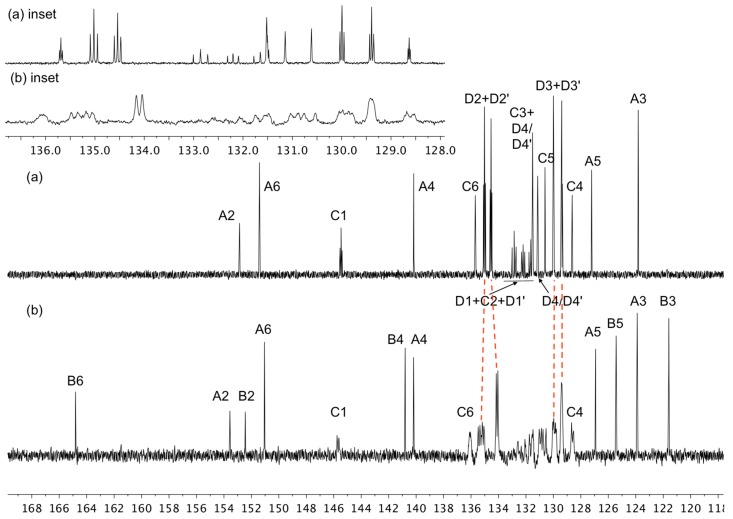
^13^C{^1^H} NMR spectra (126 MHz, acetone-*d*_6_, 298 K) of (**a**) [Cu(BIPHEP)(bpy)][PF_6_] and (**b**) [Cu(BIPHEP)(6-Etbpy)][PF_6_], with expansions of the regions *δ* 137–128 ppm (signals from C^C6^ to C^C4^), shown in the insets at the top-left. Scale: *δ*/ppm.

We have previously discussed in detail the dynamic processes observed for complexes of the types [Cu(POP)(6-Rbpy)]^+^ and [Cu(xantphos)(6-Rbpy)]^+^, where 6-Rbpy is a 6-substituted 2,2′-bipyridine [19,33]. In the ^1^H-NMR spectra in Figure 1, it is noticeable that the regions associated with the phenyl rings of the PPh_2_ units (rings labelled D) are broader for compounds containing asymmetrical 6-Mebpy and 6-Etbpy than for those with bpy or 5,5′-Me_2_bpy. The ^13^C{^1^H} NMR spectra in Figure 4 are instructive. In [Cu(BIPHEP)(bpy)][PF_6_], all signals are well-resolved and have been assigned with the aid of HMQC and HMBC spectra. The two sets of C^D1/D1’^, C^D2/D2’^, C^D3/D3’^ and C^D4/D4’^ signals arise from pairs facing towards or away from the bpy alkyl substituent (Figure 2), and the spectra are consistent with unhindered rotation of the PPh_2_ phenyl rings. Introducing the 6-alkyl substituent results in a broadening of all ^13^C{^1^H} NMR resonances arising from rings C and D (Figure 4b), consistent with increased barriers to dynamic behaviour. An inspection of the changes in line shapes of the signals for C^D2^ and C^D2’^, and for C^D3^ and C^D3^ (highlighted in Figure 4 by the red hashed lines) suggests that the energy barrier to the rotation of one set of phenyls (presumably the rings closest to the alkyl substituent) is higher than that for the second set. 

### 2.2. Single Crystal X-ray Diffraction

**Figure 5:**
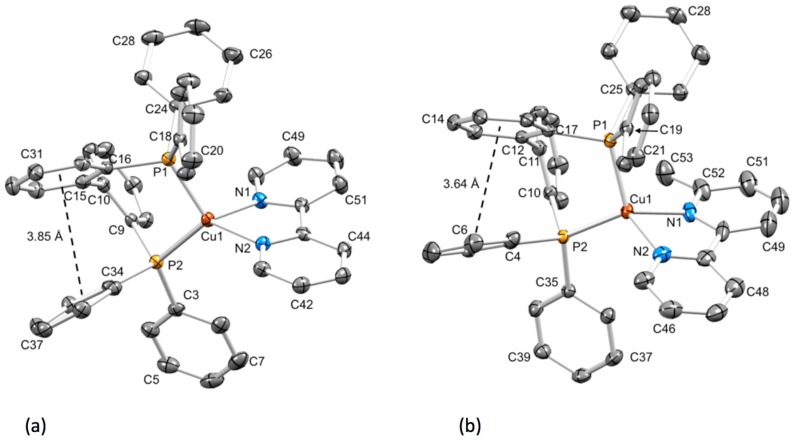
Structures of (**a**) the [Cu(BIPHEP)(bpy)]^+^ cation in [Cu(BIPHEP)(bpy)][PF_6_]∙CH_2_Cl_2_ and (**b**) the [Cu(BIPHEP)(6-Mebpy)]^+^ cation in [Cu(BIPHEP)(6-Mebpy)][PF_6_]∙Et_2_O∙0.5H_2_O. Ellipsoids are plotted at 50% probability in (**a**) and 40% in (**b**); H atoms are omitted for clarity. Each cation exhibits π-stacking between two phenyl rings in BIPHEP backbone. In (**a**) the angle between the ring planes = 25.7°, centroid...centroid distance = 3.85 Å, and in (**b**) the angle between the ring planes = 15.1°, centroid...centroid distance = 3.64 Å.

**Figure 6:**
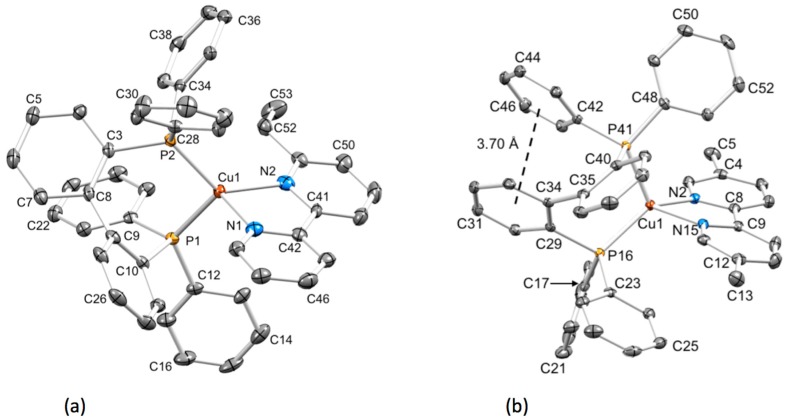
Structures of (**a**) the [Cu(BIPHEP)(6-Etbpy)]^+^ cation in [Cu(BIPHEP)(6-Etbpy)][PF_6_] and (**b**) the [Cu(BIPHEP)(5,5′-Me_2_bpy)]^+^ cation in [Cu(BIPHEP)(5,5′-Me_2_bpy)][PF_6_]∙CH_2_Cl_2_. Ellipsoids are plotted at 40% probability in (**a**) and 50% in (**b**); H atoms are omitted for clarity. For the π-stacking interaction shown in (**b**), the angle between the ring planes is 18.0° and the centroid...centroid distance is 3.70 Å.

**Figure 7:**
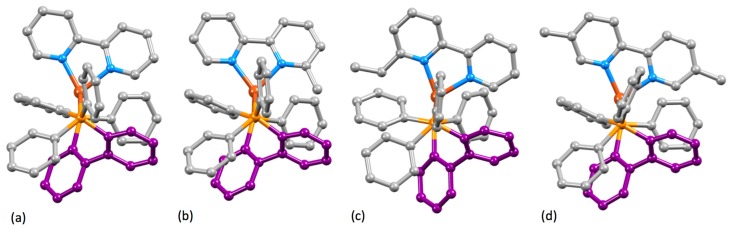
Comparisons of the distorted tetrahedral coordination spheres (viewed along the P...P vector) of copper(I) in (a) [Cu(BIPHEP)(bpy)]^+^ (τ_4_ = 0.81), (**b**) [Cu(BIPHEP)(6-Mebpy)]^+^ (τ_4_ = 0.85), (**c**) [Cu(BIPHEP)(6-Etbpy)]^+^ (τ_4_ = 0.78), and (**d**) [Cu(BIPHEP)(5,5′-Me_2_bpy)]^+^ (τ_4_ = 0.80). The origin of the chirality is the twisted backbone of BIPHEP (shown in purple).

X-ray quality crystals of [Cu(BIPHEP)(bpy)][PF_6_]∙CH_2_Cl_2_, [Cu(BIPHEP)(6-Mebpy)][PF_6_]∙Et_2_O∙0.5H_2_O, [Cu(BIPHEP)(6-Etbpy)][PF_6_] and [Cu(BIPHEP)(5,5′-Me_2_bpy)][PF_6_]∙CH_2_Cl_2_, were obtained by diffusion of Et_2_O into CH_2_Cl_2_ solutions of the respective compound. The latter two compounds crystallize in the monoclinic space groups *P*2_1_*/c* and *C*2*/c*, respectively, while [Cu(BIPHEP)(bpy)][PF_6_]∙CH_2_Cl_2_ and [Cu(BIPHEP)(6-Mebpy)][PF_6_]∙Et_2_O∙0.5H_2_O crystallize in the triclinic space group *P*−1. The solid-state structures of the [Cu(BIPHEP)(N^N)]^+^ cations are shown in Figure 5 and Figure 6, and selected structural parameters are summarized in Table 1. The twist in the backbone of the BIPHEP ligand renders each [Cu(BIPHEP)(N^N)]^+^ cation chiral (Figure 7), and both enantiomers are present in the unit cell.

The copper atoms in the complexes exhibit distorted tetrahedral geometries, with τ_4_ parameters [34] of 0.81 for [Cu(BIPHEP)(bpy)]^+^, 0.85 for [Cu(BIPHEP)(6-Mebpy)]^+^, 0.78 for [Cu(BIPHEP)(6-Etbpy)]^+^, and 0.80 for [Cu(BIPHEP)(5,5′-Me_2_bpy)]^+^. For *T*_d_ symmetry, τ_4_ = 1.00, and the values observed here indicate a distortion towards a trigonal pyramidal geometry. Figure 7 displays the four [Cu(BIPHEP)(N^N)]^+^ cations, each viewed along the P...P vector; the same enantiomer is shown for each complex. The Cu–P distances are typical (2.2248(6) to 2.2570(7) Å) and the same is true for the Cu–N distances (range = 2.0321(18) to 2.086(2) Å, Table 1). The P–Cu–P angles lie in the range 101.56(4)°–106.24(2)°. For the analogous POP and xantphos complexes with eight-membered rings, the bite angle of the coordinated phosphanes is around 113° for the complexes with 6-Mebpy or 6-Etbpy [18,19], and in the range 111.97(3)°–115.00(3)° for [Cu(POP)(bpy)][PF_6_] and [Cu(xantphos)(bpy)][PF_6_] [15,16,17]. As expected, the chelating 2,2′-bipyridine ligands exhibit similar N–Cu–N angles, in the range 79.75(14)°–80.65(10)°. The two rings of the bpy ligand are twisted in all the complex cations, with N–C–C–N torsion angles between 6.4(5)° in [Cu(BIPHEP)(6-Etbpy)]^+^, and −12.1(4)° in [Cu(BIPHEP)(6-Mebpy)]^+^.

There are no trends in the structural parameters as a function of the number or steric demands of the alkyl substituents attached to the bpy unit, and it appears that packing effects are dominant. In the complexes containing [Cu(BIPHEP)(bpy)][PF_6_]^+^, [Cu(BIPHEP)(6-Mebpy)]^+^ and [Cu(BIPHEP)(5,5′-Me_2_bpy)]^+^, intracation π-stacking interactions occur between a phenyl ring of a PPh_2_ group and one ring of the BIPHEP backbone (Figure 5 and Figure 6b, and Figure S18 in the Supporting Information) although the angles between the ring planes of 25.7°, 15.1° and 18.0° mean that the interactions are not optimal. Short C–H...F cation...anion contacts feature in all of the structures, and the accommodation of solvent molecules in [Cu(BIPHEP)(bpy)][PF_6_]∙CH_2_Cl_2_, [Cu(BIPHEP)(6-Mebpy)][PF_6_]∙Et_2_O∙0.5H_2_O and [Cu(BIPHEP)(5,5′-Me_2_bpy)][PF_6_]∙CH_2_Cl_2_ also contributes to packing effects.

### 2.3. Electrochemical Behaviour

The electrochemical behaviour of the [Cu(BIPHEP)(N^N)][PF_6_] compounds was investigated using cyclic voltammetry (CV), and cyclic voltammograms for CH_2_Cl_2_ solutions are shown in Figures S19–S22 (see Supporting Information). Each compound exhibits a reversible or quasi-reversible oxidation process (Table 2) assigned to a Cu^+^/Cu^2+^ oxidation, but no ligand-based reduction processes were observed within the solvent-accessible window. For comparison, Table 2 also reports the oxidation potentials of [Cu(P^P)(bpy)][PF_6_] and [Cu(P^P)(6-Mebpy)][PF_6_] (P^P = POP and xantphos) [17,18], measured under the same conditions as the BIPHEP-containing compounds. For the BIPHEP complexes, the oxidation potentials increase on going from the complex with the unsubstituted bpy (+0.59 V), to 6-Mebpy (+0.72 V) and 6-Etbpy (+0.73 V). This follows the trend in the corresponding xantphos-containing compounds (Table 2), although, with the more flexible POP ligand, the introduction of the 6-methyl substituent has little effect. The trends for the BIPHEP-containing compounds are consistent with the introduction of the 6-alkyl substituent, hindering the flattening of the tetrahedral copper(I) towards square planar copper(II), thereby moving the oxidation to a higher potential. The Cu^+^/Cu^2+^ oxidation occurs at similar potentials in [Cu(BIPHEP)(5,5′-Me_2_bpy)][PF_6_] and [Cu(BIPHEP)(bpy)][PF_6_] (Table 2), indicating that the presence of methyl groups in these positions has little stabilizing effect on the copper(I) oxidation state. The data in Table 2 reveal that metal oxidation in [Cu(BIPHEP)(bpy)][PF_6_] occurs at significantly lower potential than in [Cu(POP)(bpy)][PF_6_] and [Cu(xantphos)(bpy)][PF_6_], suggesting that the flattening of the copper coordination sphere that accompanies oxidation from Cu^+^ to Cu^2+^ occurs more easily. This should also be reflected in the excited state lifetimes, which are discussed in the next section. We note that there is no clear correlation between the Cu^+^/Cu^2+^ oxidation potential in solution and the τ_4_ value (see Section 2.2), which gives a measure of the distortion away from a tetrahedral geometry (for which τ_4_ = 1.00) in the solid state. 

### 2.4. Photophysical Properties

**Figure 8:**
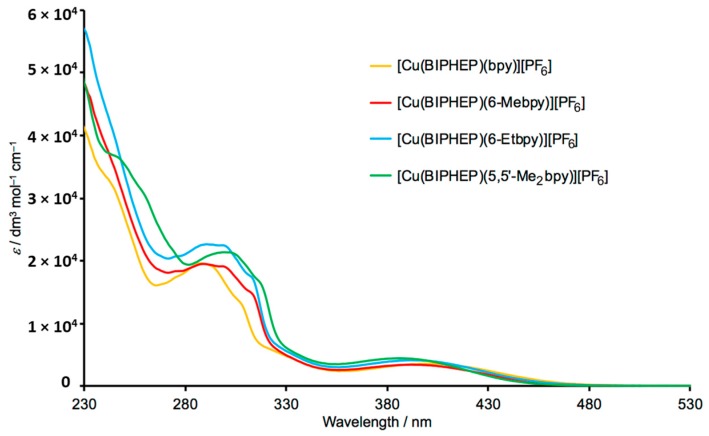
Solution absorption spectra of the [Cu(BIPHEP)(N^N)][PF_6_] complexes (CH_2_Cl_2_, 3.5–5.3 × 10^−5^ mol dm^−3^).

The solution absorption spectra of the [Cu(BIPHEP)(N^N)][PF_6_] compounds in CH_2_Cl_2_ are displayed in Figure 8 and absorption maxima are given in Table 3. The spectra for all four compounds are similar, with intense, high-energy (230–330 nm) absorptions arising from spin-allowed, ligand-based π*←π transitions. The broad absorption, with *λ*_max_ close to 400 nm, is assigned to the MLCT band, and values of *λ*_max_ and *ε*_max_ vary little across the series of compounds.

**Figure 9:**
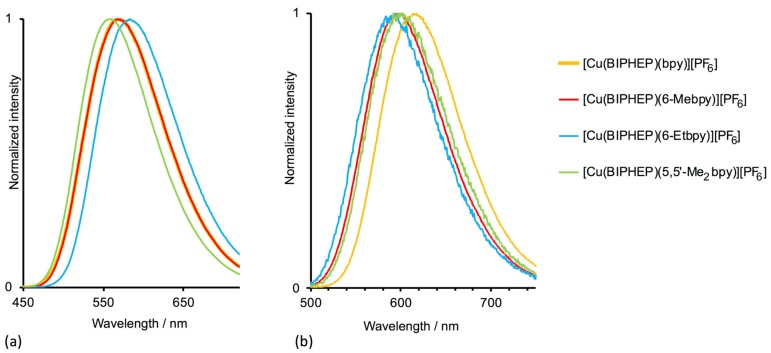
(**a**) Normalized emission spectra of the powdered [Cu(BIPHEP)(N^N)][PF_6_] compounds, *λ*_exc_ = 365 nm. The curves for [Cu(BIPHEP)(bpy)][PF_6_] and [Cu(BIPHEP)(6-Mebpy)][PF_6_] are virtually superimposed. (**b**) Normalized emission spectra of the [Cu(BIPHEP)(N^N)][PF_6_] complexes in frozen Me-THF at 77 K. *λ*_exc_ = 410 nm.

When excited into the MLCT band at 390 nm, deaerated solutions of [Cu(BIPHEP)(bpy)][PF_6_] did not show a detectable emission, while [Cu(BIPHEP)(6-Mebpy)][PF_6_], [Cu(BIPHEP)(6-Etbpy)][PF_6_] and [Cu(BIPHEP)(5,5′-Me_2_bpy)][PF_6_] exhibited weak emissions, with values of *λ*_em_^max^ = 645, 636 and 641 nm, respectively (Figure S23 in Supporting Information). Since these emissions were so weak, we focused on solid-state and frozen-glass emission behaviour. Emission maxima, photoluminescence quantum yields (PLQY) and excited state lifetimes (*τ*_1/2_) are presented in Table 4. Powdered samples are yellow emitters with values of *λ*_em_^max^, in the range 558–583 nm (Figure 9a), and are similar to those observed for [Cu(P^P)(bpy)][PF_6_], [Cu(P^P)(6-Mebpy)][PF_6_] and [Cu(P^P)(6-Etbpy)][PF_6_], in which P^P is POP or xantphos [17,18,19]. A blue shift in the emission maximum, upon going from liquid solution to a solid state, is consistent with previous observations of these families of compounds [19,22]. The highest PLQY for the solid-state compounds (Table 4) is 14% for [Cu(BIPHEP)(5,5′-Me_2_bpy)][PF_6_] and this also shows the longest excited state lifetime (8 μs). This may indicate better protection of the copper centre against quenchers (e.g., O_2_ and H_2_O) [22], although substitution in the 5,5′-positions, in the case of [Cu(POP)(5,5′-(CF_3_)_2_bpy)][PF_6_] and [Cu(xantphos)(5,5′-(CF_3_)_2_bpy)][PF_6_], proved less beneficial than a 6,6′-substitution pattern [17]. As noted earlier, we were not able to isolate [Cu(BIPHEP)(6,6′-Me_2_bpy)][PF_6_] and are not, therefore, able to compare the effects of going from 5,5′-Me_2_bpy to 6,6′-Me_2_bpy in the case of the BIPHEP co-ligand. 

In order to investigate whether the [Cu(BIPHEP)(N^N)][PF_6_] compounds were TADF emitters, their emission and excited state lifetimes were recorded at low temperature and compared to their values at room temperature. Since the repopulation of the singlet excited state by reverse intersystem crossing is suppressed at low temperatures (Δ*E* > *kT*), the excited state lifetime should be considerably longer, since phosphorescence from the triplet excited state should be the dominant process. The normalized emission spectra of the [Cu(BIPHEP)(N^N)][PF_6_] complexes in frozen Me-THF at 77 K are depicted in Figure 9b, and the emission maxima and excited state lifetimes are summarized in Table 4. A comparison of the values of *λ*_em_^max^ in the powders at room temperature with those at 77 K reveals that the emission maxima are red-shifted for all the complexes, consistent with the triplet excited state being lower in energy than the singlet excited state. A significant extension of the excited state lifetimes is observed (Table 4), and, for the compounds in which the bpy bears alkyl substituents, values of *τ*_1/2_ are ca. 50 μs. These data are consistent with [Cu(BIPHEP)(N^N)][PF_6_] (N^N = 6-Mebpy, 6-Etbpy and 5,5′-Me_2_bpy) being TADF emitters. The increased excited state lifetime on going from 298 to 77 K is particularly noticeable for [Cu(BIPHEP)(6-Etbpy)][PF_6_], where *τ*_1/2_ increases from 1 μs at room temperature to 53 μs at 77 K. At the same time, the temperature-induced red-shift in this complex is the smallest in the series, and at 77 K, it has an emission maximum at the shortest wavelength compared to others in the series. This raises the question as to whether other effects such as non-radiative decay processes occur in [Cu(BIPHEP)(6-Etbpy)][PF_6_].

Since [Cu(BIPHEP)(5,5′-Me_2_bpy)][PF_6_] exhibited the highest PLQY and longest excited state lifetime in the solid state, we decided to conduct preliminary tests in LECs. Simple bilayer devices were fabricated by spin-coating a solution of [Cu(BIPHEP)(5,5′-Me_2_bpy)][PF_6_] on indium tin oxide-coated glass substrates, covered with a film of poly(3,4-ethylenedioxythiophene) polystyrene sulfonate. Devices were finished with the thermal evaporation of an aluminium cathode. LECs exhibited yellow electroluminescence with *λ*_em_^max^ = 586 nm, red-shifted with respect to the solid-state photoluminescence. However, even at a current density of 100 A m^−2^, the maximum luminance was as low as 12 cd m^−2^, with a maximum external quantum efficiency (EQE) of 0.03%. The LEC also showed only a short lifetime, with *t*_1/2_ = 0.1 hours. In comparison, LECs containing [Cu(xantphos)(6-Mebpy)][PF_6_], [Cu(xantphos)(6-Etbpy)][PF_6_] and [Cu(POP)(6-Etbpy)][PF_6_] in the emissive layers exhibit values of *t*_1/2_ greater than 15, 40 and 80 h, respectively [18,19]. 

## 3. Materials and Methods 

### 3.1. General

^1^H and ^13^C{^1^H} and ^31^P-NMR spectra were recorded on a Bruker Avance III-500 spectrometer (Bruker BioSpin AG, Fällanden, Switzerland) at 298 K. ^1^H and ^13^C-NMR chemical shifts were referenced, with respect to residual solvent peaks (*δ* TMS = 0), and ^31^P-NMR chemical shifts, with respect to *δ*(85% aqueous H_3_PO_4_) = 0 ppm. Solution absorption and emission spectra were measured using a Shimadzu UV-2600 spectrophotometer and a Shimadzu RF-5301PC spectrofluorometer, respectively (Shimadzu Schweiz GmbH, 4153 Aesch, Switzerland). A Shimadzu LCMS-2020 instrument (Shimadzu Schweiz GmbH, 4153 Aesch, Switzerland) was used to record electrospray ionization (ESI) mass spectra; samples were introduced as MeCN solutions with added formic acid. 

Quantum yields (CH_2_Cl_2_ solutions or powder samples) were measured with a Hamamatsu absolute photoluminescence quantum yield spectrometer C11347 Quantaurus-QY (Hamamatsu Photonics, 4500 Solothurn, Switzerland). Emission lifetimes and powder emission spectra were recorded using a Hamamatsu Compact Fluorescence lifetime Spectrometer C11367 Quantaurus-Tau (Hamamatsu Photonics), with an LED light source with *λ*_exc_ = 365 nm. Low temperature emission and lifetime measurements were made using an Edinburgh Instruments LP920-KS instrument (Edinburgh Instruments, Livingston, UK), in frozen 2-methyloxolane (Me-THF); a 410 nm excitation was obtained from pulsed third-harmonic radiation from a Quantel Brilliant b Nd:YAG laser (Quantal Laser, Lumibird, 22300 Lannion, France), equipped with a Rainbow optical parameter oscillator. The laser pulse duration and pulse frequency were ~10 ns and 10 Hz, respectively, with a typical pulse energy of 7 mJ. An iCCD camera (Andor, Oxford Instruments GmbH, Wiesbaden, Germany) was used for detection of the spectra. Single-wavelength kinetics were recorded using a photomultiplier tube. 

6-Methyl-2,2′-bipyridine (6-Mebpy) and 6-ethyl-2,2′-bipyridine (6-Etbpy) [35] and [Cu(MeCN)_4_][PF_6_] [36] were prepared following the literature procedures. BIPHEP was bought from Alfa Aesar (Alfa Aesar GmbH, 76185 Karlsruhe, Germany), and 2,2′-bipyridine (bpy), 5,5′-dimethyl-2,2′-bipyridine (5,5′-Me_2_bpy) and 6,6′-dimethyl-2,2′-bipyridine (6,6′-Me_2_bpy) from Sigma-Aldrich (Merck, 9470 Buchs, Switzerland). All chemicals were used as received.

### 3.2. [Cu(BIPHEP)(bpy)][PF_6_]

A colourless solution of bpy (39 mg, 0.25 mmol) and BIPHEP (131 mg, 0.25 mmol) in CH_2_Cl_2_ (20 mL) was added to a colourless solution of [Cu(MeCN)_4_][PF_6_] (93 mg, 0.25 mmol) in CH_2_Cl_2_ (20 mL), turning the solution yellow. After stirring at room temperature for 2 h, the solvent was removed in vacuo. The crude product was redissolved in CH_2_Cl_2_ and [Cu(BIPHEP)(bpy)][PF_6_] (63 mg, 0.071 mmol, 28%), precipitated as a yellow powder by layering with Et_2_O. ^1^H-NMR (500 MHz, acetone-*d*_6_, 298 K): *δ*/ppm 8.76 (dt, *J* = 8.2, 1.0 Hz, 2H, H^A3^), 8.42 (dd, *J* = 5.2, 1.4 Hz, 2H, H^A6^), 8.29 (td, *J* = 7.9, 1.6 Hz, 2H, H^A4^), 7.67 (m, 2H, H^A5^), 7.42–7.37 (m, 2H, H^D4/D4^^’^), 7.34–7.26 (overlapping m, 16H, H^D2+D2’+D3/D3^^’+D4/D4’+C5^), 7.26–7.17 (overlapping m, 6H, H^D3/D3^^’+C4^), 7.10 (m, 2H, H^C6^), 6.81 (m, 2H, H^C3^). ^13^C{^1^H} NMR (126 MHz, acetone-*d*_6_, 298 K): *δ*/ppm 152.9 (t, *J*_PC_ = 2 Hz, C^A2^), 151.4 (C^A6^), 146.5 (t, *J*_PC_ = 9 Hz, C^C1^), 140.2 (C^A4^), 135.7 (t, *J*_PC_ = 3.5 Hz, C^C6^), 135.0 (t, *J*_PC_ = 8 Hz, C^D2/D2^^’^), 134.5 (t, *J*_PC_ = 8 Hz, C^D2/D2^^’^), 132.9 (t, *J*_PC_ = 18 Hz, C^D1/D1^^’^), 132.2 (t, *J*_PC_ = 14 Hz, C^C2^), 131.6 (t, *J*_PC_ = 18 Hz, C^D1/D1^^’^), 131.6 (C^C3^), 131.5 (t, *J*_PC_ = 1 Hz, C^D4/D4^^’^), 131.2 (t, *J*_PC_ = 1 Hz, C^D4/D4^^’^), 130.6 (C^C5^), 130.0 (t, *J*_PC_ = 5 Hz, C^D3/D3^^’^), 129.4 (t, *J*_PC_ = 5 Hz, C^D3/D3^^’^), 128.6 (t, *J*_PC_ = 3 Hz, C^C4^), 127.2 (C^A5^), 123.8 (C^A3^). ^31^P{^1^H} NMR (202 MHz, acetone-*d*_6_, 298 K): *δ*/ppm −1.2 (broad, FWHM = 270 Hz, BIPHEP), −142.5 (septet, *J*_PF_ = 700 Hz, [PF_6_]^−^). ESI MS: *m/z* 741.10 [M–PF_6_]^+^ (base peak, calc. 741.16). Found C 61.53, H 4.37, N 3.22; C_46_H_36_CuF_6_N_2_P_3_∙H_2_O requires C 61.03, H 4.23, N 3.09.

### 3.3. [Cu(BIPHEP)(6-Mebpy)][PF_6_]

A colourless solution of 6-Mebpy (43 mg, 0.25 mmol) and BIPHEP (131 mg, 0.25 mmol) in CH_2_Cl_2_ (20 mL) was added to a colourless solution of [Cu(MeCN)_4_][PF_6_] (93 mg, 0.25 mmol) in CH_2_Cl_2_ (20 mL), turning the solution yellow. After stirring at room temperature for 2 h, the solvent was removed under reduced pressure. The crude product was redissolved in CH_2_Cl_2_ and layered with Et_2_O, to precipitate [Cu(BIPHEP)(6-Mebpy)][PF_6_] (202 mg, 0.23 mmol, 92%) as a yellow powder. ^1^H-NMR (500 MHz, acetone-*d*_6_, 298 K): *δ*/ppm 8.72 (dt, *J* = 8.3, 1.0 Hz, 1H, H^A3^), 8.62 (d, *J* = 7.9 Hz, 1H, H^B3^), 8.29–8.21 (overlapping m, 2H, H^B4+A4^), 8.04 (dt, *J* = 5.2, 0.8 Hz, 1H, H^A6^), 7.73 (dd, *J* = 7.8, 0.9 Hz, 1H, H^B5^), 7.54 (m, 1H, H^A5^), 7.46–7.10 (overlapping m, 26H, H^D2+D2’+D3+D3’+D4+D4’+C4+C4’+C5+C5’+C6+C6’^), 6.99 (m, 1H, H^C3/C3^^’^), 6.88 (m, 1H, H^C3/C3^^’^), 2.58 (s, 3H, H^Me^). ^13^C{^1^H} NMR (126 MHz, acetone-*d*_6_, 298 K): *δ*/ppm 160.5 (C^B6^), 153.5 (C^A2/B2^), 152.7 (C^A2/B2^), 151.0 (C^A6^), 145.7 (br, C^C1+C1^^’^), 140.6 (C^A4/B4^), 140.2 (C^A4/B4^), 136.2 (br, C^C6/C6^^’^), 135.2 (br, overlapping, C^D2/D2’^), 134.2 (C^D2/D2’^), 134.1 (C^D2/D2’^), 129.45 (C^D3/D3’^), 129.4 (C^D3/D3’^), 128.8 (br, C^C4+C4’^), 127.6 (C^B5^), 127.0 (C^A5^), 123.9 (C^A3^), 121.4 (C^B3^); signals for C^C3^, C^C5^, C^C1^, C^C2^, C^D1^ were poorly resolved. ^31^P{^1^H} NMR (202 MHz, acetone-*d*_6_, 298 K): *δ*/ppm −1.3 (broad, FWHM = 428 Hz, BIPHEP) −3.7 (broad, FWHM = 401 Hz, BIPHEP), −144.2 (septet, *J*_PF_ = 700 Hz, [PF_6_]^−^). ESI MS: *m/z* 755.16 [M–PF_6_]^+^ (base peak, calc. 755.18). Found C 61.89, H 4.83, N 3.38; C_47_H_38_CuF_6_N_2_P_3_∙H_2_O requires C 61.41, H 4.39, N 3.05.

### 3.4. [Cu(BIPHEP)(6-Etbpy)][PF_6_]

A solution of 6-Etbpy (46 mg, 0.25 mmol) and BIPHEP (131 mg, 0.25 mmol) in CH_2_Cl_2_ (20 mL) was added to a solution of [Cu(MeCN)_4_][PF_6_] (93 mg, 0.25 mmol) in CH_2_Cl_2_ (20 mL), causing an immediate change from colourless to yellow. After stirring at room temperature for 2 h, the solvent was removed in vacuo. The crude material was redissolved in CH_2_Cl_2_. Layering with Et_2_O precipitated [Cu(BIPHEP)(6-Etbpy)][PF_6_] (208 mg, 0.23 mmol, 92%) as a yellow powder. ^1^H-NMR (500 MHz, acetone-*d*_6_, 298 K): *δ*/ppm 8.72 (d, *J* = 8.2 Hz, 1H, H^A3^), 8.62 (d, *J* = 7.8 Hz, 1H, H^B3^), 8.31 (t, *J* = 7.9 Hz, 1H, H^B4^), 8.24 (td, *J* = 7.6, 1.6 Hz, 1H, H^A4^), 7.91 (m, 1H, H^A6^), 7.79 (d, *J* = 7.7 Hz, 1H, H^B5^), 7.51 (m, 1H, H^A5^), 7.42–7.12 (overlapping m, 25H, H^D4+D4’+C5+C5’+D2+D2’+D3+D3’+C4+C4’+C6/C6’^), 7.08 (m, 1H, C^C6/C6’^), 7.00 (m, 1H, H^C3^), 6.88 (m, 1H, H^C3^), 3.03 (q, *J* = 7.8 Hz, 2H, H^Et^), 1.08 (t, *J* = 7.6 Hz, 3H, H^Et^). ^13^C{^1^H} (126 MHz, acetone-*d*_6_, 298 K): 164.8 (C^B6^), 153.6 (C^A2/B2^), 152.4 (C^A2/B2^), 151.0 (C^A6^), 145.8 (C^C1/C1’^), 145.6 (C^C1/C1’^), 140.8 (C^B4^), 140.2 (C^A4^), 136.1 (C^C6+C6’^), 135.3 (overlapping m, C^D2/D2’^), 134.2 (C^D2/D2’^), 134.0 (C^D2/D2’^), 132.7–131.6 (overlapping m, C^D1+D1’+C2+C2’^), 131.5 (br, C^C3+C3’^), 131.0 (C^D4/D4’^), 130.9 (C^D4/D4’^), 130.8 (C^D4/D4’^), 130.5 (C^D4/D4’^), 130.0 and 129.8 (br, overlapping m, C^D3/D3’+C5+C5’^), 129.4 (br, overlapping m, C^D3/D3’^), 128.7 (br, C^C4/C4’^), 128.5 (br, C^C4/C4’^), 126.9 (C^A5^), 125.4 (C^B5^), 123.9 (C^A3^), 121.6 (C^B3^), 35.5 (C^Et^), 13.2 (C^Et^); see text discussion. ^31^P{^1^H} NMR (162 MHz, acetone-*d*_6_, 300 K) *δ*/ppm −1.3 (broad, FWHM = 320 Hz, BIPHEP), −4.0 (broad, FWHM = 320 Hz, BIPHEP), −144.2 (septet, *J*_PF_ = 708 Hz, [PF_6_]^−^); see text discussion. ESI MS: *m/z* 769.28 [M–PF_6_]^+^ (base peak, calc. 769.20). Found C 62.40, H 4.98, N 3.22; C_48_H_40_CuF_6_N_2_P_3_∙0.5H_2_O requires C 62.37, H 4.47, N 3.03.

### 3.5. [Cu(BIPHEP)(5,5′-Me_2_bpy)][PF_6_]

A solution of 5,5′-Me_2_bpy (46 mg, 0.25 mmol) and BIPHEP (131 mg, 0.25 mmol) in CH_2_Cl_2_ (20 mL) was added to a solution of [Cu(MeCN)_4_][PF_6_] (93 mg, 0.25 mmol) in CH_2_Cl_2_ (20 mL). The yellow reaction mixture was stirred at room temperature for 2 h, and then solvent was removed under reduced pressure. The crude product was redissolved in CH_2_Cl_2_ and layering with Et_2_O led to the precipitation of yellow [Cu(BIPHEP)(5,5′-Me_2_bpy)][PF_6_] (215 mg, 0.23 mmol, 92%). ^1^H-NMR (500 MHz, acetone-*d*_6_, 298 K): *δ*/ppm ^1^H-NMR (500 MHz, acetone-*d*_6_, 298 K) *δ* /ppm 8.55 (d, *J* = 8.2 Hz, 2H, H^B3^), 8.04 (m, 2H, H^B4^), 7.96 (m, 2H, H^B6^), 7.44–7.39 (m, 2H, H^D4/D4’^), 7.38–7.29 (overlapping m, 12H, H^D2/D2’+D3/D3’+D4/D4’+C5^), 7.27–7.19 (m, 10H, H^D2/D2’+D3/D3’+C4^), 7.15 (m, 2H, H^C6^), 6.76 (m, 2H, H^C3^), 2.30 (s, 6H, H^Me^). ^13^C{^1^H} NMR (126 MHz, acetone-*d*_6_, 298 K) δ /ppm 151.5 (C^B6^), 150.5 (t, *J* = 2 Hz, C^B2^), 145.7 (t, *J* = 10 Hz, C^C1^), 140.2 (C^B4^), 137.3 (C^B5^), 135.8 (t, *J* = 4 Hz, C^C6^), 135.2 (t, *J* = 9 Hz, C^D2/D2’^), 134.6 (t, *J* = 8 Hz, C^D2/D2’^), 132.8 (t, *J* = 18 Hz, C^D1/D1’^), 132.2 (t, *J* = 14 Hz, C^C2^), 131.7 (t, *J* = 3 Hz, C^C3^), 131.6 (C^D4/D4’^), 131.3 (t, *J* = 18 Hz, C^D1/D1’^), 131.1 (C^D4/D4’^), 130.6 (C^C5^), 130.0 (t, *J* = 5 Hz, C^D3/D3’^), 129.3 (t, *J* = 5 Hz, C^D3/D3’^), 128.6 (t, *J* = 3 Hz, C^C4^), 122.8 (C^B3^), 18.1 (C^Me^). ^31^P{^1^H} NMR (162 MHz, acetone-*d*_6_, 300 K) *δ*/ppm −0.6 (broad, FWHM = 350 Hz, BIPHEP), −144.2 (septet, *J*_PF_ = 708 Hz, [PF_6_]^−^). ESI MS: *m/z* 769.28 [M–PF_6_]^+^ (base peak, calc. 769.20). Found C 58.24, H 4.30, N 3.02; C_48_H_40_CuF_6_N_2_P_3_∙CH_2_Cl_2_ requires C 58.84, H 4.23, N 2.80.

### 3.6. Crystallography

Single crystal X-ray diffraction data were collected on a Bruker Kappa Apex2 diffractometer with data reduction, solution and refinement, using the programs APEX [37] and CRYSTALS [38]. Structural analysis used the program Mercury v. 4.1.2 [39,40]. 

### 3.7. [Cu(BIPHEP)(bpy)][PF_6_]∙CH_2_Cl_2_

C_47_H_38_Cl_2_CuF_6_N_2_P_3_, *M* = 972.19, yellow plate, monoclinic, space group *P*2_1_/*c*, *a* = 14.3304(6), *b* = 15.4136(6), *c* = 20.6025(7) Å, *β* = 105.844(2)°, *U* = 4377.8(3) Å^3^, *Z* = 4, *D_c_* = 1.475 Mg m^−3^, *μ*(Cu-Kα) = 3.397 mm^−1^, *T* = 123 K. Total 46,656 reflections, 8189 unique, *R*_int_ = 0.071. Refinement of 6109 reflections (550 parameters) with *I* >2σ(*I*) converged at final *R*_1_ = 0.0505 (*R*_1_ all data = 0.0733), *wR*_2_ = 0.1151 (*wR*_2_ all data = 0.1308), gof = 0.9710. CCDC 1958102.

### 3.8. [Cu(BIPHEP)(6-Mebpy)][PF_6_]∙Et_2_O∙0.5H_2_O 

C_51_H_49_CuF_6_N_2_O_1.5_P_3_, *M* = 984.42, yellow block, monoclinic, space group *C*2*/c*, *a* = 29.5271(19), *b* = 16.7361(11), *c* = 20.5516(14) Å, *β* = 111.249(2)°, *U* = 9465.5(11) Å^3^, *Z* = 8, *D_c_* = 1.381 Mg m^−3^, *μ*(Cu-Kα) = 2.157 mm^−1^, *T* = 123 K. Total 31,108 reflections, 8537 unique, *R*_int_ = 0.023. Refinement of 8430 reflections (584 parameters) with *I* >2σ(*I*) converged at final *R*_1_ = 0.0540 (*R*_1_ all data = 0.0544), *wR*_2_ = 0.1197 (*wR*_2_ all data = 0.1197), gof = 1.0146. CCDC 1958100.

### 3.9. [Cu(BIPHEP)(6-Etbpy)][PF_6_] 

C_48_H_40_CuF_6_N_2_P_3_, *M* = 915.31, yellow block, triclinic, space group *P*−1, *a* = 10.5819(11), *b* = 13.4147(14), *c* = 15.0134(16) Å, *α* = 93.766(3) *β* = 100.826(3), γ = 92.405(3)°, *U* = 2085.5(4) Å^3^, *Z* = 2, *D_c_* = 1.457 Mg m^−3^, *μ*(Cu-Kα) = 2.377 mm^−1^, *T* = 123 K. Total 29,365 reflections, 7594 unique, *R*_int_ = 0.032. Refinement of 6965 reflections (541 parameters) with *I* >2σ(*I*) converged at final *R*_1_ = 0.0412 (*R*_1_ all data = 0.0502), *wR*_2_ = 0.0822 (*wR*_2_ all data = 0.1385), gof = 1.3893. CCDC 1958101.

### 3.10. [Cu(BIPHEP)(5,5′-Me_2_bpy)][PF_6_]∙CH_2_Cl_2_

C_49_H_42_Cl_2_CuF_6_N_2_P_3_, *M* = 1000.25, yellow block, triclinic, space group *P*−1, *a* = 10.9543(6), *b* = 10.9953(6), *c* = 18.8408(11) Å, *α* = 94.9723(17), *β* = 92.9633(18), *γ* = 96.7453(17)°, *U* = 2240.7(2) Å^3^, *Z* = 2, *D_c_* = 1.482 Mg m^−3^, *μ*(Cu-Kα) = 3.334 mm^−1^, *T* = 123 K. Total 29,278 reflections, 7850 unique, *R*_int_ = 0.030. Refinement of 7808 reflections (568 parameters) with *I* >2σ(*I*) converged at final *R*_1_ = 0.0453 (*R*_1_ all data = 0.0453), *wR*_2_ = 0.0461 (*wR*_2_ all data = 0.0461), gof = 1.1280. CCDC 1958099.

## 4. Conclusions

We have prepared and characterized the compounds [Cu(BIPHEP)(bpy)][PF_6_], [Cu(BIPHEP)(6-Mebpy)][PF_6_], [Cu(BIPHEP)(6-Etbpy)][PF_6_] and [Cu(BIPHEP)(5,5′-Me_2_bpy)][PF_6_]. ^1^H, ^13^C{^1^H} and ^31^P{^1^H} NMR spectra, including the use of 2D methods, reveal dynamic processes involving the BIPHEP ligand. The single crystal structures of [Cu(BIPHEP)(bpy)][PF_6_]∙CH_2_Cl_2_, [Cu(BIPHEP)(5,5′-Me_2_bpy)][PF_6_]∙CH_2_Cl_2_, [Cu(BIPHEP)(6-Mebpy)][PF_6_]∙Et_2_O∙0.5H_2_O and [Cu(BIPHEP)(6-Etbpy)][PF_6_] were determined and confirmed distorted tetrahedral {Cu(P^P)(N^N)} coordination environments. Each compound shows a quasi-reversible Cu^+^/Cu^2+^ process, which moves to a higher potential upon the introduction of 6-methyl or 6-ethyl substituent into the bpy unit. The [Cu(BIPHEP)(N^N)][PF_6_] compounds are weak emitters in the deaerated solution, whereas powdered samples are yellow emitters at room temperature, with values of *λ*_em_^max^ between 558 and 583 nm, and PLQY values up to 14%. Evidence that the compounds with N^N = 6-Mebpy, 6-Etbpy and 5,5′-Me_2_bpy exhibit TADF comes from the fact that their excited state lifetimes increase from *τ*_1/2_ < 8 μs to *τ*_1/2_ values of up to 53 μs on cooling from room temperature to 77 K, and that the emission maxima are red-shifted.

## Figures and Tables

**Table 1:** Selected structural parameters for the [Cu(BIPHEP)(N^N][PF_6_] compounds.

Cation in [Cu(BIPHEP)(N^N)][PF_6_]	Cu–P Distance/Å	Cu–N distance/Å	P–Cu–P Angle/Deg	N–Cu–N Angle/Deg	N–C–C–N Torsion Angle/Deg
[Cu(BIPHEP)(bpy)]^+^	Cu1–P1 = 2.2415(10); Cu1–P2 = 2.2292(9)	Cu1–N1 = 2.071(3); Cu1–N2 = 2.040(3)	P2–Cu1–P1 = 104.70(4)	N2–Cu1–N1 = 80.15(11)	−11.7(5)
[Cu(BIPHEP)(6-Mebpy)]^+^	Cu1–P1 = 2.2570(7); Cu1–P2 = 2.2549(7)	Cu1–N1 = 2.086(2); Cu1–N2 = 2.049(2)	P2–Cu1–P1 = 105.01(2)	N1–Cu1–N2 = 80.65(10)	−12.1(4)
[Cu(BIPHEP)(6-Etbpy)]^+^	Cu1–P1 = 2.2410(11); Cu1–P2 = 2.2322(10)	Cu1–N1 = 2.072(4); Cu1–N2 = 2.063(4)	P2–Cu1–P1 = 101.56(4)	N2–Cu1–N1 = 79.75(14)	6.4(5)
[Cu(BIPHEP)(5,5′-Me_2_bpy)]^+^	Cu1–P16 = 2.2248(6); Cu1–P41 = 2.2515(6)	Cu1–N2 = 2.0321(18); Cu1–N15 = 2.0751(18)	P16–Cu1–P41 = 106.24(2)	N2–Cu1–N15 = 80.51(7)	−10.3(3)

**Table 2:** Cyclic voltammetric data for [Cu(BIPHEP)(N^N)][PF_6_] complexes referenced to internal Fc/Fc^+^ = 0.0 V; CH_2_Cl_2_ (dried) solutions, with [*^n^*Bu_4_N][PF_6_] as the supporting electrolyte and a scan rate of 0.1 V s^−1^. Processes are quasi-reversible.

Cation in [Cu(BIPHEP)(N^N][PF_6_]	$E_{1/2}^{ox}$/V	*E*_pc_ – *E*_pa_/mV
[Cu(BIPHEP)(bpy)]^+^	+0.59	100
[Cu(BIPHEP)(6-Mebpy)]^+^	+0.72	129
[Cu(BIPHEP)(6-Etbpy)]^+^	+0.73	133
[Cu(BIPHEP)(5,5′-Me_2_bpy)]^+^	+0.57	197
[Cu(POP)(bpy)]^+^	+0.72 ^a^	110 ^a^
[Cu(POP)(6-Mebpy)]^+^	+0.69 ^b^	-
[Cu(xantphos)(bpy)]^+^	+0.76 ^a^	110 ^a^
[Cu(xantphos)(6-Mebpy)]^+^	+0.90 ^a^	100 ^a^

^a^ Data taken from reference [17]. ^b^ Data taken from reference [18].

**Table 3:** Solution absorption maxima for the [Cu(BIPHEP)(N^N)][PF_6_] complexes (CH_2_Cl_2_ with solution concentrations in the range 3.5–5.3 × 10^−5^ mol dm^−3^).

Cation in [Cu(BIPHEP)(N^N)][PF_6_]	*λ*_max_/nm (*ε*/dm^3^ mol^−1^ cm^−1^)	
	Ligand-Based π*←π	MLCT
[Cu(BIPHEP)(bpy)]^+^	244 sh (32,000), 291 (19,400), 309 sh (12,400)	407 (3500)
[Cu(BIPHEP)(6-Mebpy)]^+^	292 (19,400), 301 (18,500), 316 sh (14,500)	400 (3400)
[Cu(BIPHEP)(6-Etbpy)]^+^	292 (22,500), 301 (22,100), 315 sh (15,800)	400 (4000)
[Cu(BIPHEP)(5,5′-Me_2_bpy)]^+^	248 sh (36,200), 301 (21,300), 319 sh (15,200)	390 (4400)

**Table 4:** Emission maxima, photoluminescence quantum yields (PLQY) and lifetimes (*τ*_1/2_) for [Cu(BIPHEP)(N^N)][PF_6_] complexes.

Cation in [Cu(BIPHEP)(N^N)][PF_6_]	Powder ^a^			Frozen Glass (Me-THF, 77 K) ^b^	
	*λ*_em_^max^/nm ^c^	PLQY/%	*τ*_1/2_/μs	*λ*_em_^max^/nm ^d^	*τ*_1/2_/μs
[Cu(BIPHEP)(bpy)]^+^	566	4	3	615	7
[Cu(BIPHEP)(6-Mebpy)]^+^	568	7	3	595	45
[Cu(BIPHEP)(6-Etbpy)]^+^	583	3	1	590	53
[Cu(BIPHEP)(5,5′-Me_2_bpy)]^+^	558	14	8	600	49

^a^*λ*_exc_ = 365 nm; ^b^ *λ*_exc_ = 410 nm. ^c^ *λ*_em_^max^ estimated error ± 2 nm. ^d^ *λ*_em_^max^ estimated error ± 5 nm.

## Supplementary Materials

The following are available online. Crystallographic data (cifs) for the four structures. Figures S1–S4: mass spectra; Figures S5–S16: 2D NMR spectra; Figure S17: 31P{1H} NMR spectrum of [Cu(BIPHEP)(6-Etbpy)][PF6]; Figure S18: structural diagrams to illustrate π-stacking interactions in [Cu(BIPHEP)(bpy)]^+^, [Cu(BIPHEP)(6-Mebpy)]^+^ and [Cu(BIPHEP)(5,5′-Me2bpy)]^+^; Figures S19–S22: cyclic voltammograms; Figure S23: solution emission spectra.
