# Supplementary material for: Hinged and Wide: A New P^P Ligand for Emissive [Cu(P^P)(N^N)][PF6] Complexes
Source: Molecules. 2019 Oct 31;24(21):3934. doi: 10.3390/molecules24213934 (PMC6864541; doi:10.3390/molecules24213934)
Supplement: Supplementary file 1 [file molecules-24-03934-s001.pdf]

Supporting Information to accompany:

Hinged and wide: a new P^P ligand for emissive $[Cu(P^{\wedge}P)(N^{\wedge}N)][PF_6]$ complexes

Sarah Keller [1], Matthias Bantle [1], Alessandro Prescimone [1], Edwin C. Constable [1] and Catherine E. Housecroft [1*]

[1] Department of Chemistry, University of Basel, BPR 1096, Mattenstrasse 24a, CH-4058 Basel, Switzerland; sarah.keller@chimieparistech.psl.eu; mattu.bantle@gmail.com; alessandro.prescimone@unibas.ch; edwin.constable@unibas.ch

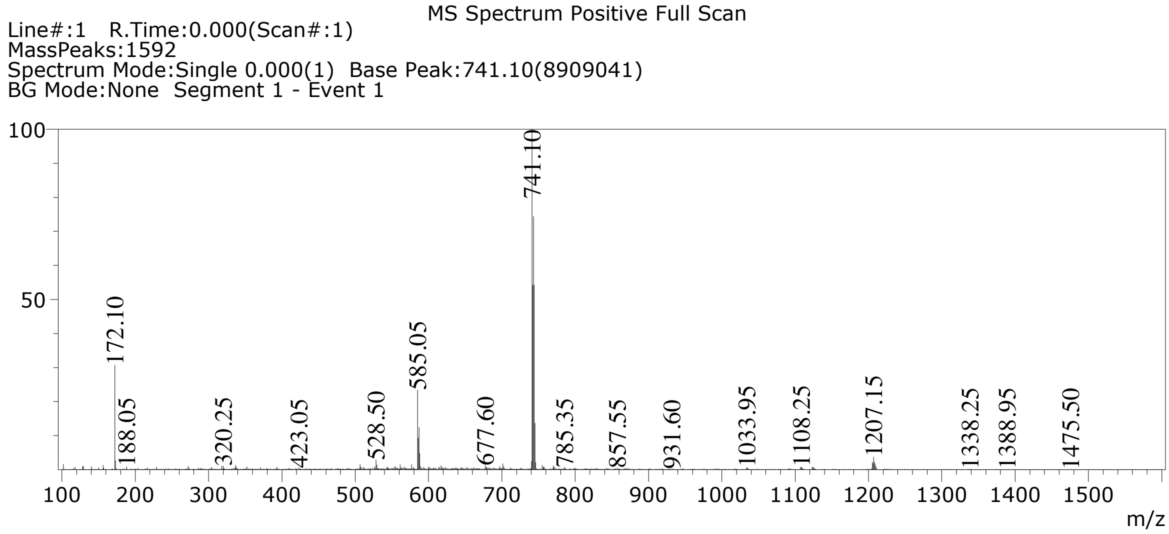

Figure S1. Electrospray mass spectrum (positive mode) of $[Cu(BIPHEP)(bpy)][PF_6]$.

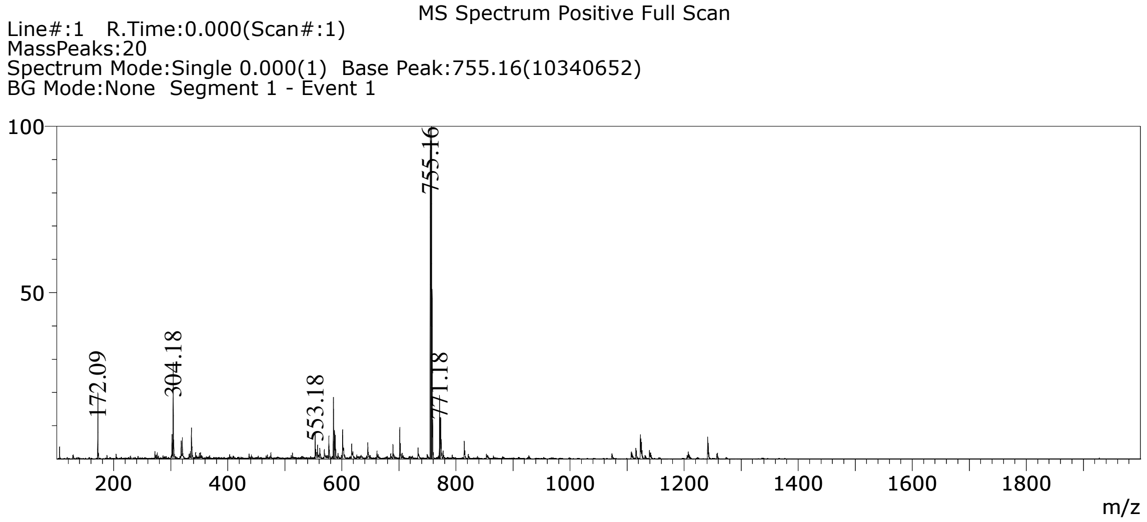

Figure S2. Electrospray mass spectrum (positive mode) of $[Cu(BIPHEP)(6\text{-}Mebpy)][PF_6]$.

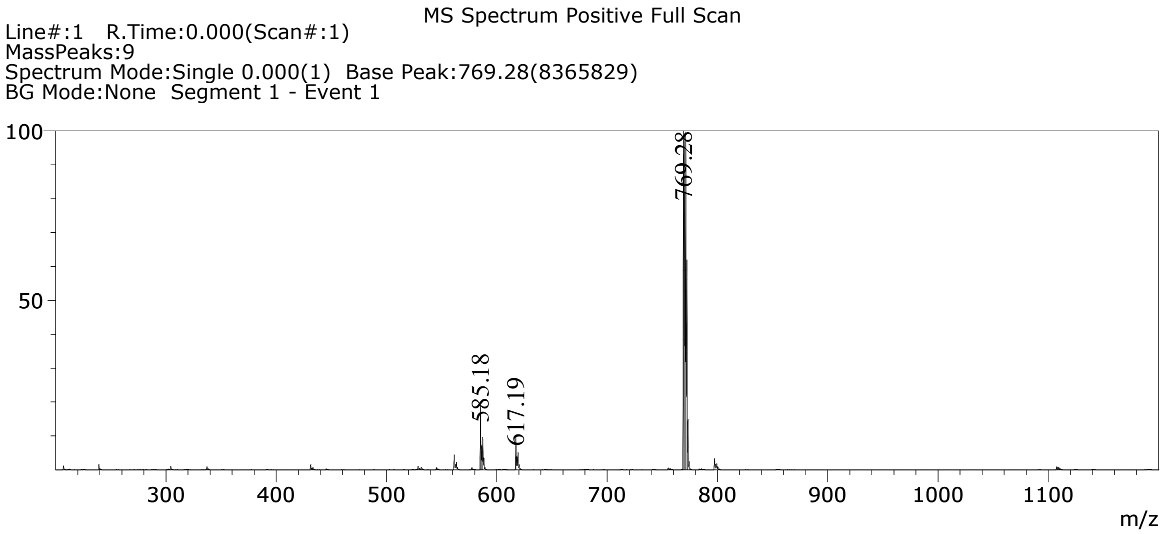

Figure S3. Electrospray mass spectrum (positive mode) of [Cu(BIPHEP)(6-Etbpy)][PF_6].

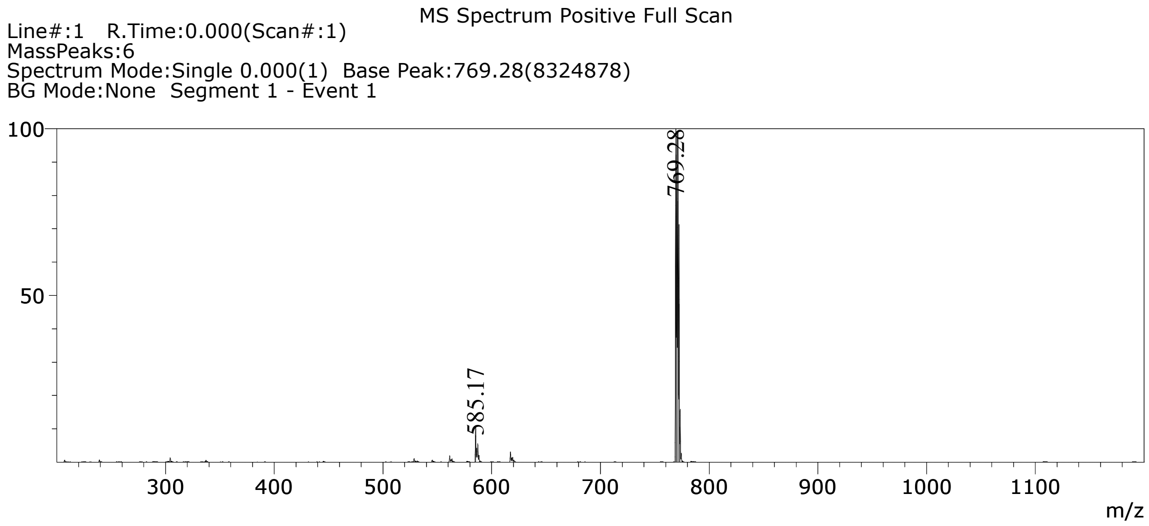

Figure S4. Electrospray mass spectrum (positive mode) of [Cu(BIPHEP)(5,5'-Me_2bpy)][PF_6].

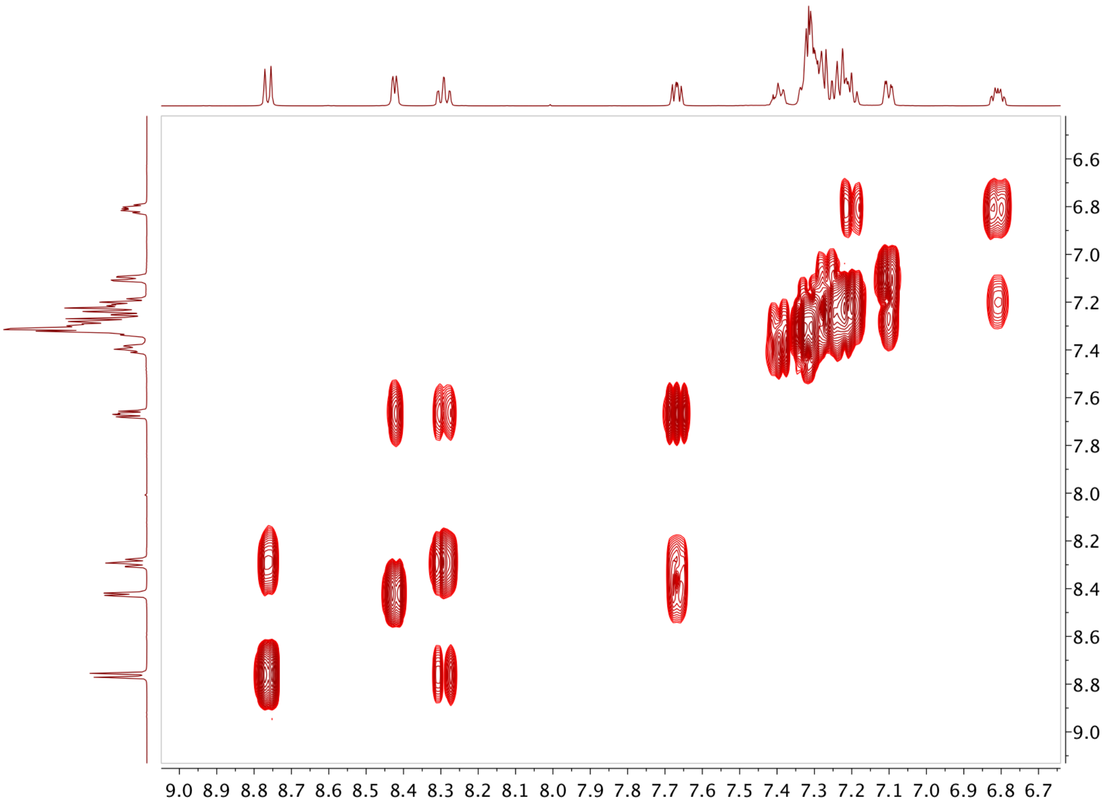

Figure S5. COSY spectrum of [Cu(BIPHEP)(bpy)][PF_6] (500 MHz, acetone-d_6, 298 K). Scales: δ / ppm.

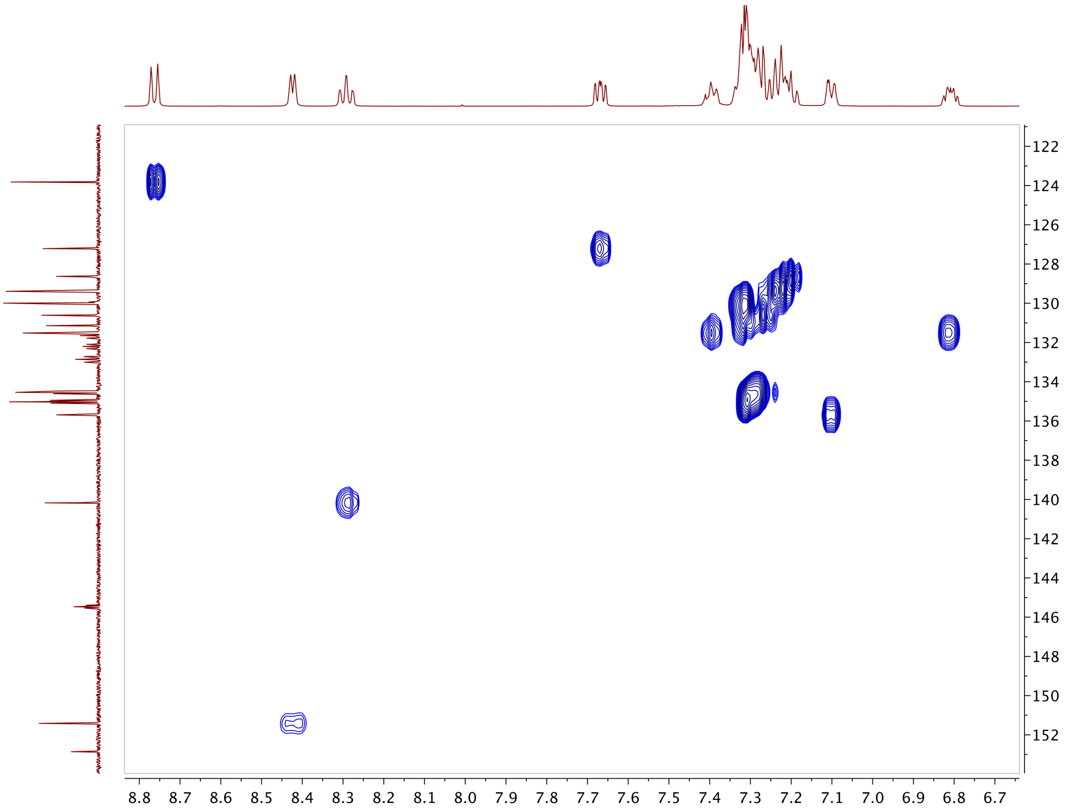

Figure S6. HMQC spectrum of [Cu(BIPHEP)(bpy)][PF_6] (^{1}H 500 MHz, ^{13}C{^{1}H} 126 MHz, acetone-d_6, 298 K). Scales: δ / ppm.

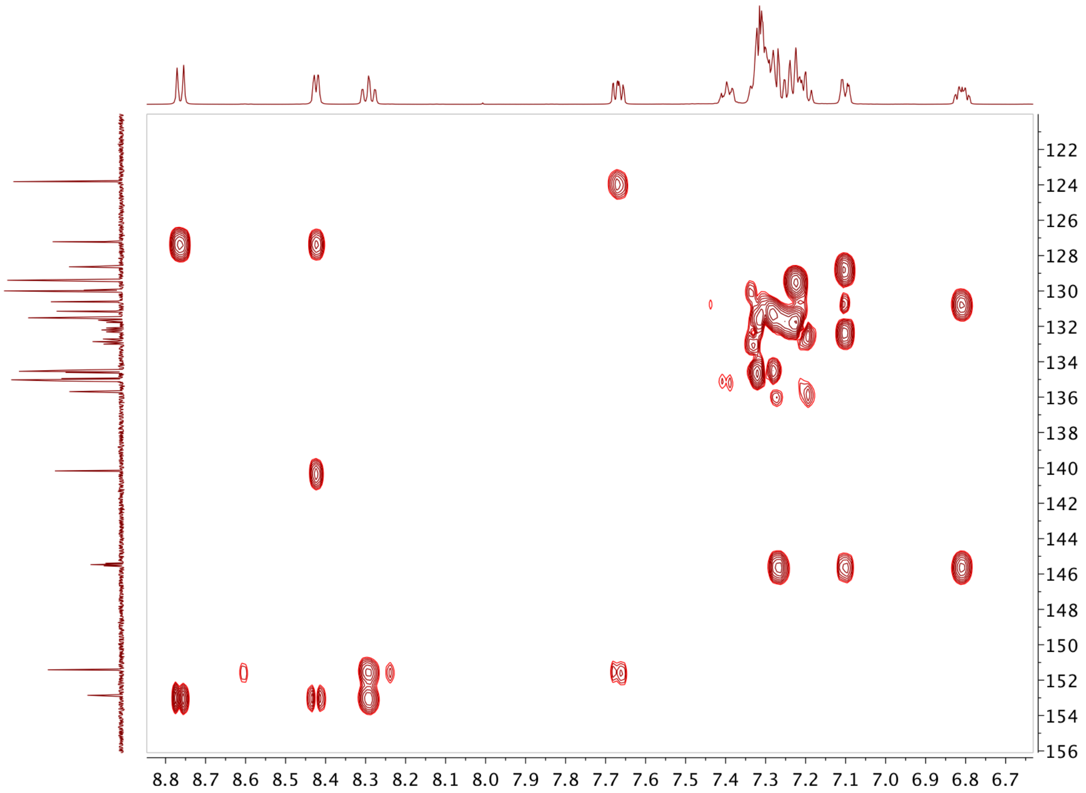

Figure S7. HMBC spectrum of [Cu(BIPHEP)(bpy)][PF_6] (^{1}H 500 MHz, $^{13}C\{^{1}H\}$ 126 MHz, acetone-d_6, 298 K). Scales: δ / ppm.

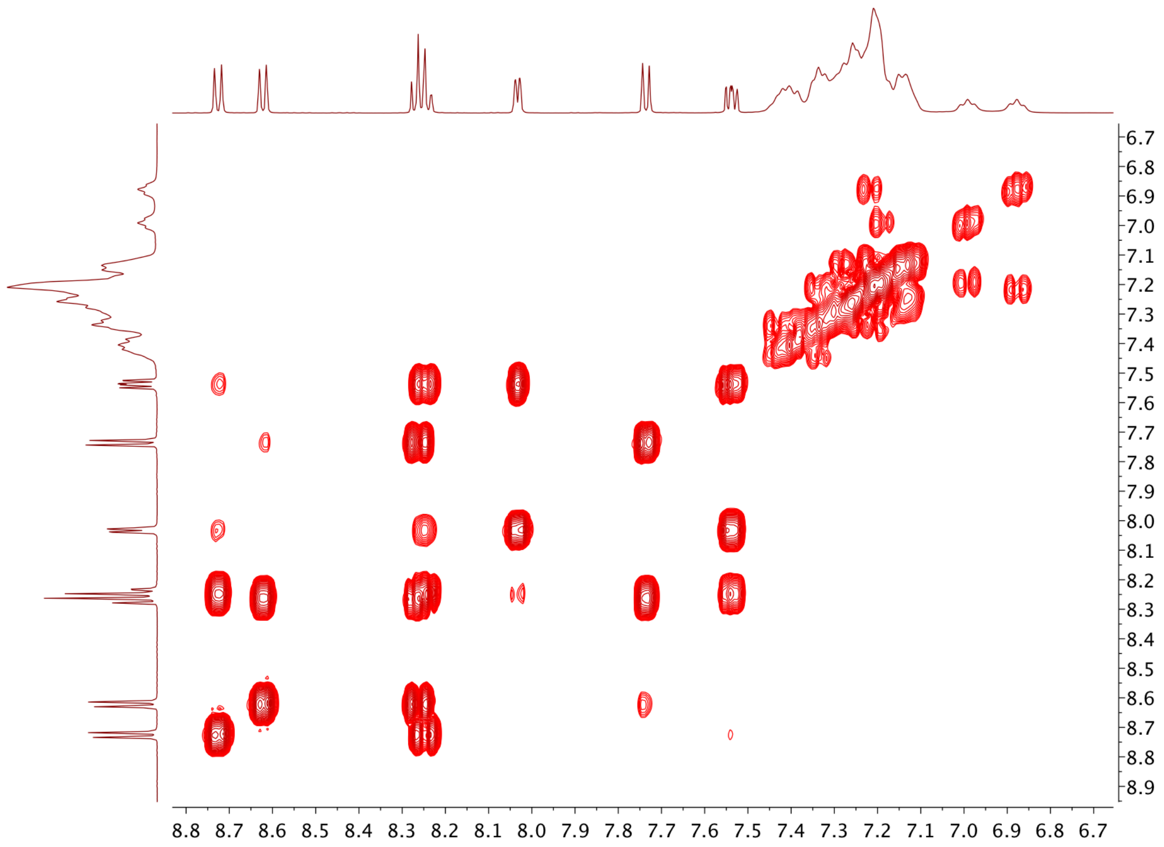

Figure S8. COSY spectrum of [Cu(BIPHEP)(6-Mebpy)][PF_6] (500 MHz, acetone-d_6, 298 K). Scales: δ / ppm.

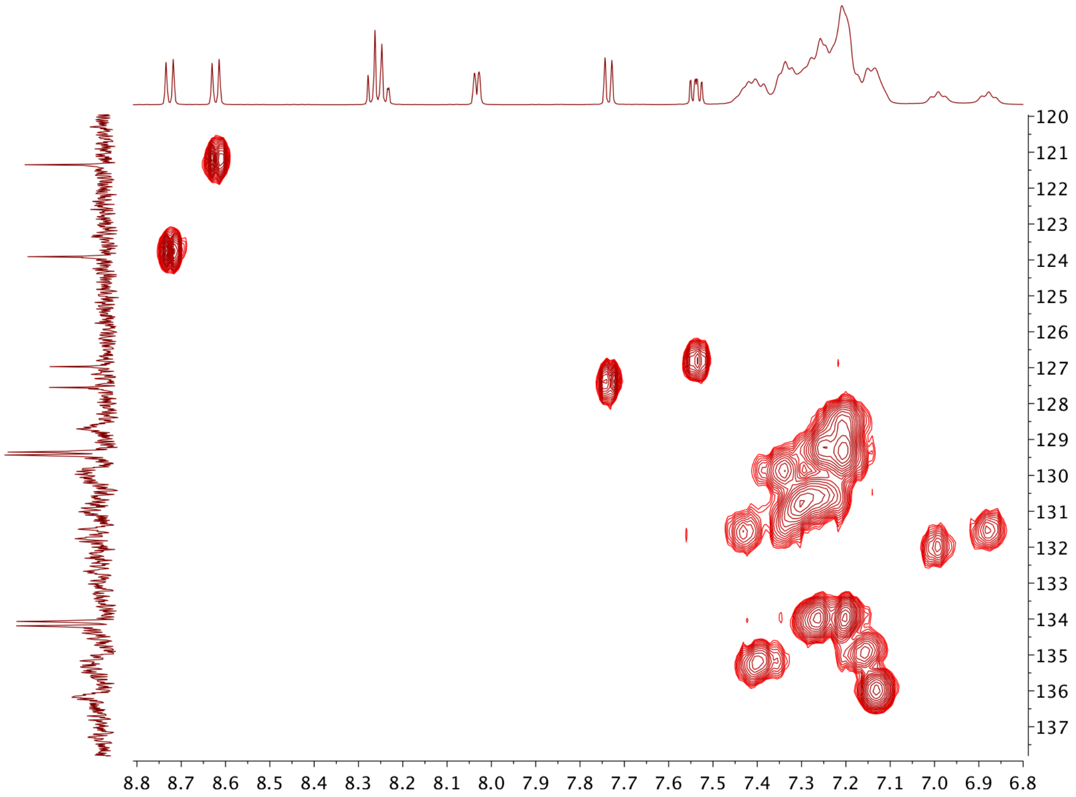

Figure S9. HMQC spectrum of [Cu(BIPHEP)(6-Mebpy)][PF_6] (^{1}H 500 MHz, ^{13}C{^{1}H} 126 MHz, acetone-d_6, 298 K). Scales: δ / ppm.

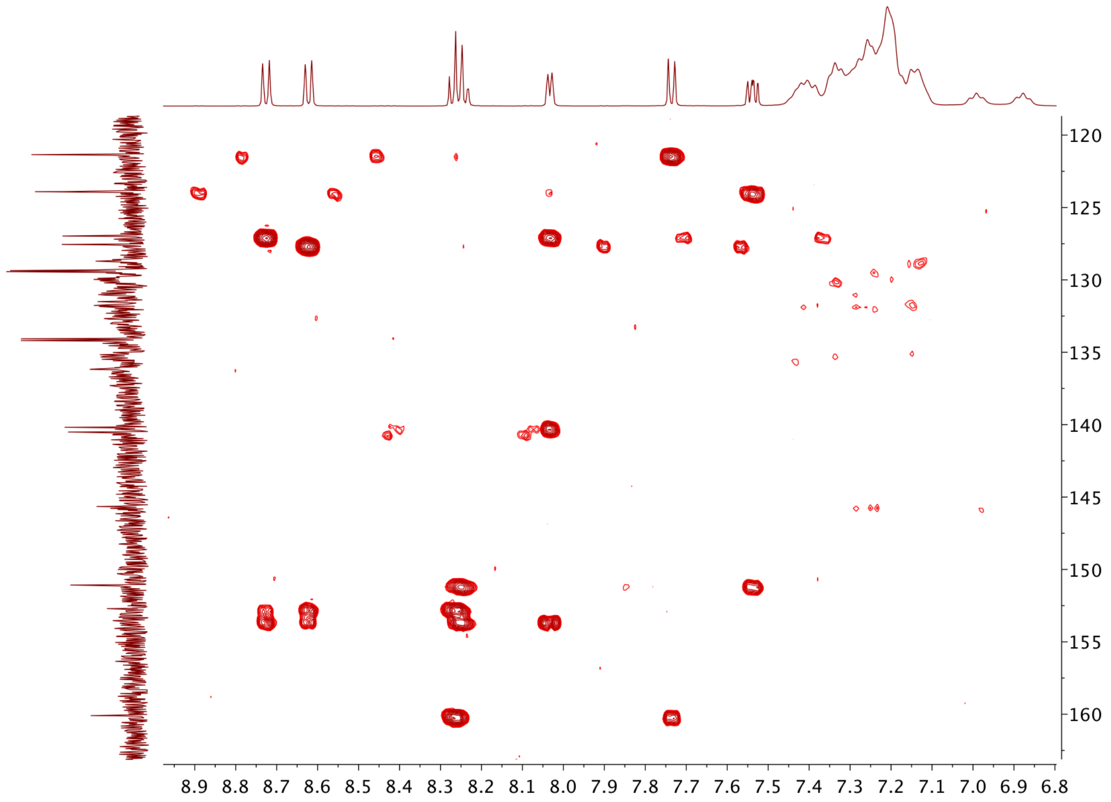

Figure S10. HMBC spectrum of [Cu(BIPHEP)(6-Mebpy)][PF_6] (^{1}H 500 MHz, ^{13}C{^{1}H} 126 MHz, acetone-d_6, 298 K). Scales: δ / ppm.

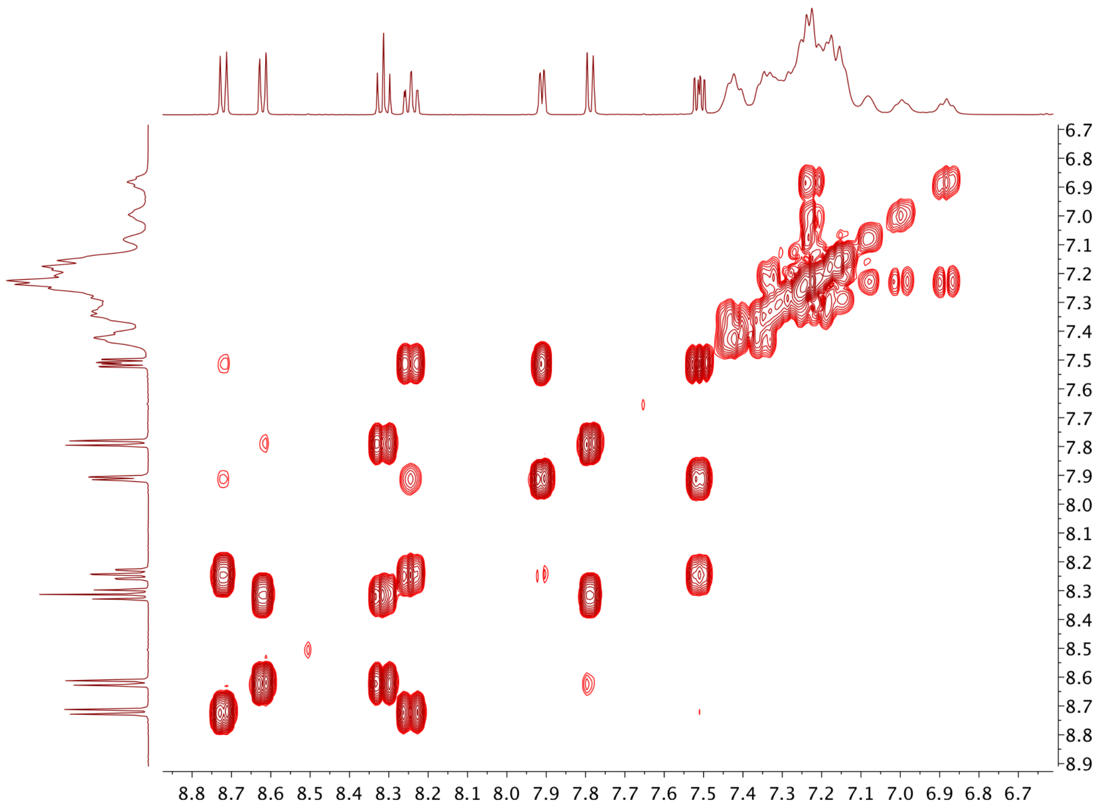

Figure S11. COSY spectrum of [Cu(BIPHEP)(6-Etbpy)][PF_6] (500 MHz, acetone-d_6, 298 K). Scales: δ / ppm.

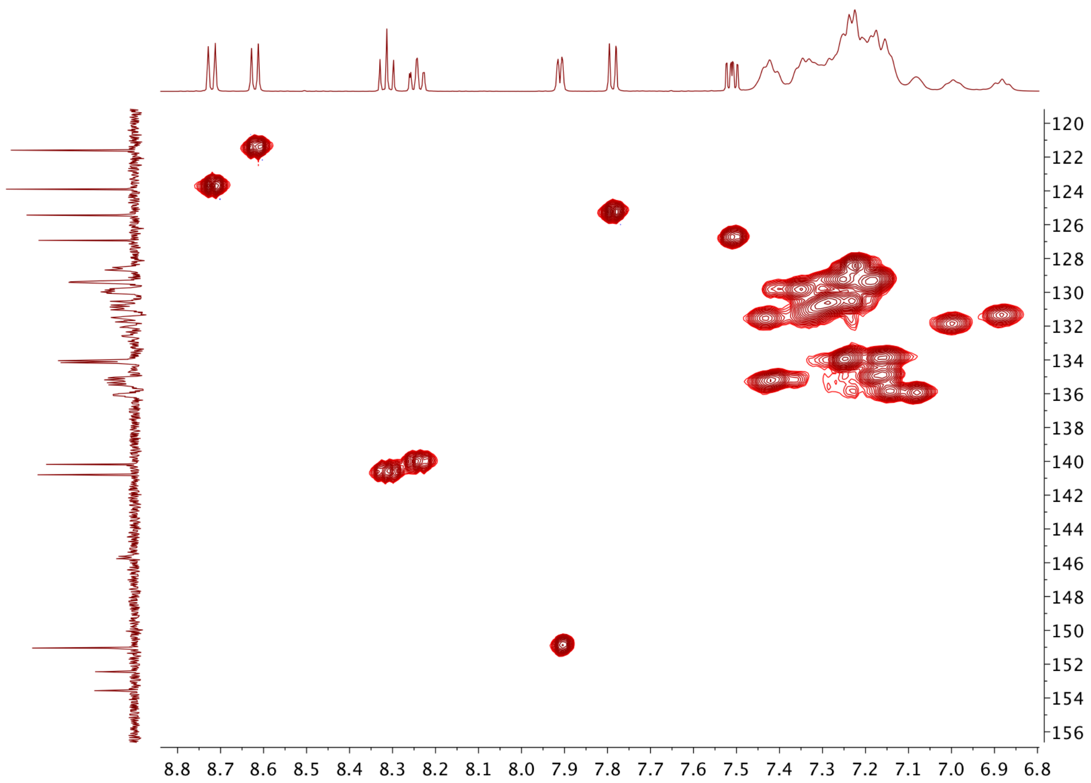

Figure S12. HMQC spectrum of [Cu(BIPHEP)(6-Etbpy)][PF_6] (^1H 500 MHz, $^{13}C\{^1H\}$ 126 MHz, acetone-d_6, 298 K). Scales: δ / ppm.

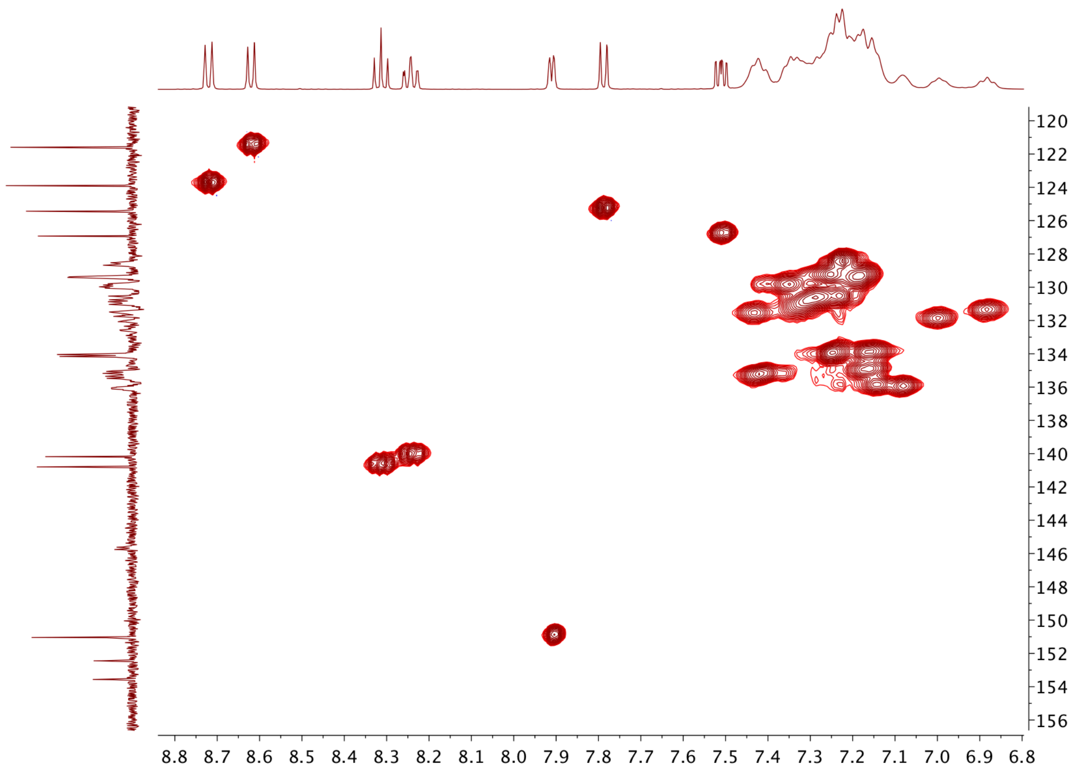

Figure S13. HMBC spectrum of [Cu(BIPHEP)(6-Etbpy)][PF_6] (^{1}H 500 MHz, ^{13}C{^{1}H} 126 MHz, acetone-d_6, 298 K). Scales: δ / ppm.

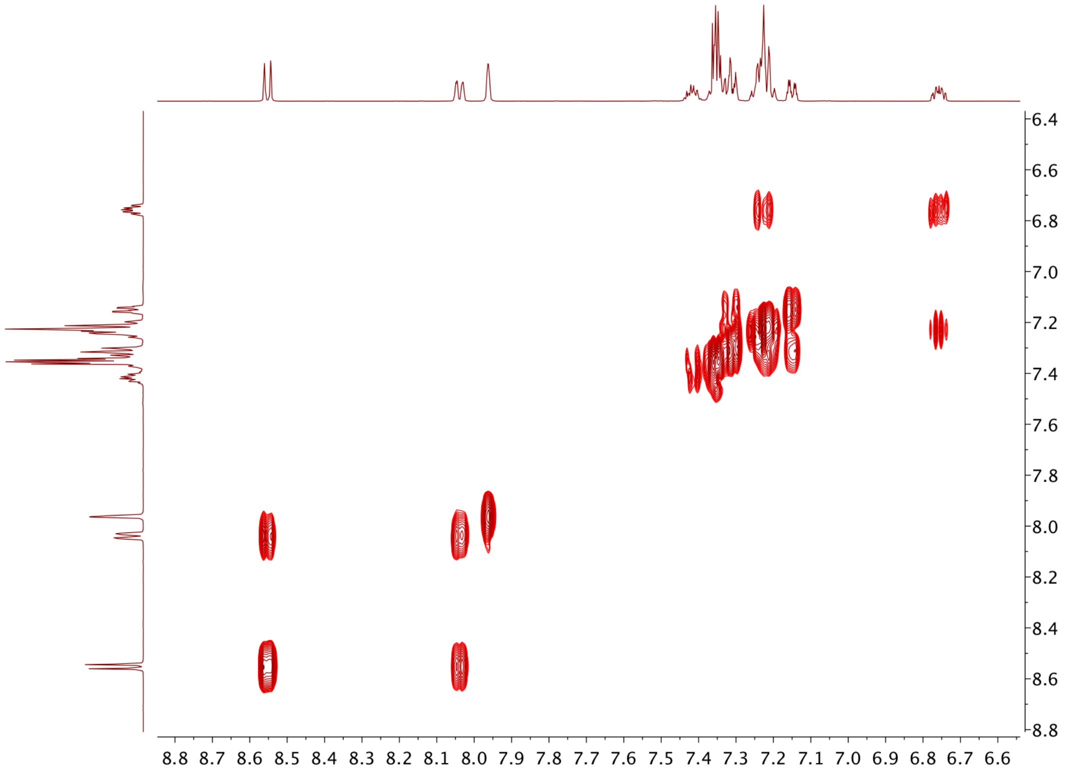

Figure S14. COSY spectrum of [Cu(BIPHEP)(5,5'-Me_2bpy)][PF_6] (500 MHz, acetone-d_6, 298 K). Scales: δ / ppm.

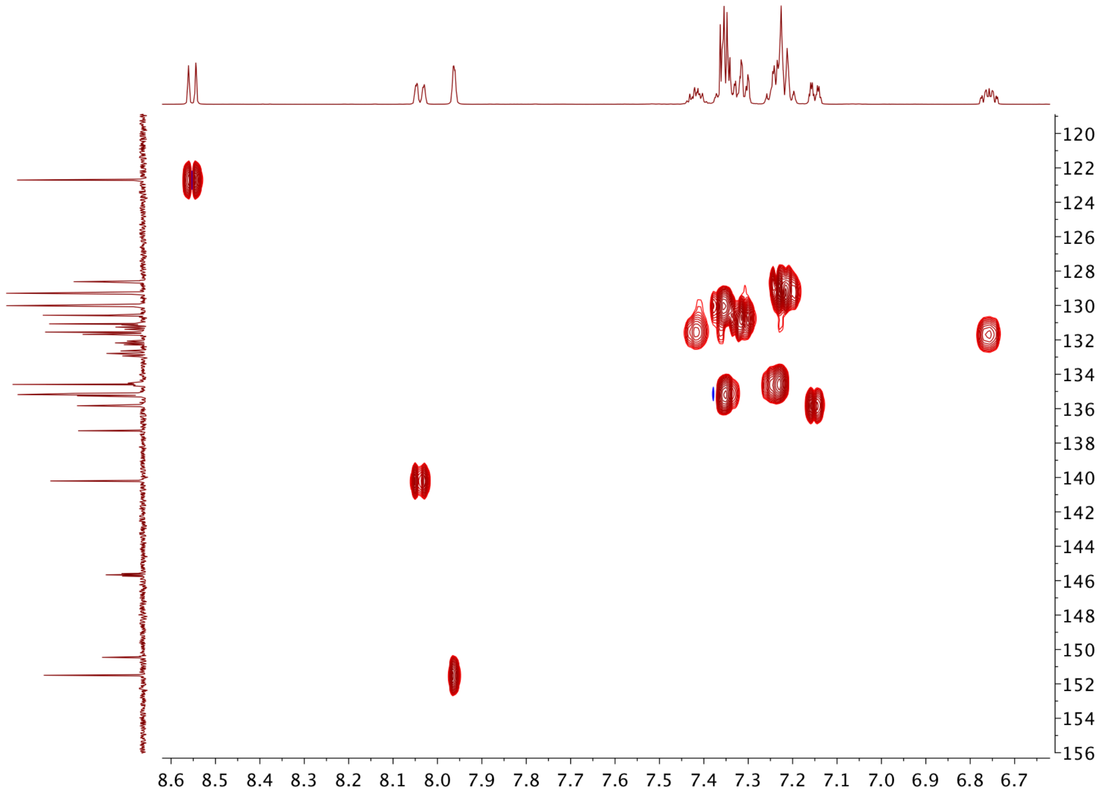

Figure S15. HMQC spectrum of [Cu(BIPHEP)(5,5'-Me_2bpy)][PF_6] (^{1}H 500 MHz, ^{13}C{^{1}H} 126 MHz, acetone-d_6, 298 K). Scales: δ / ppm.

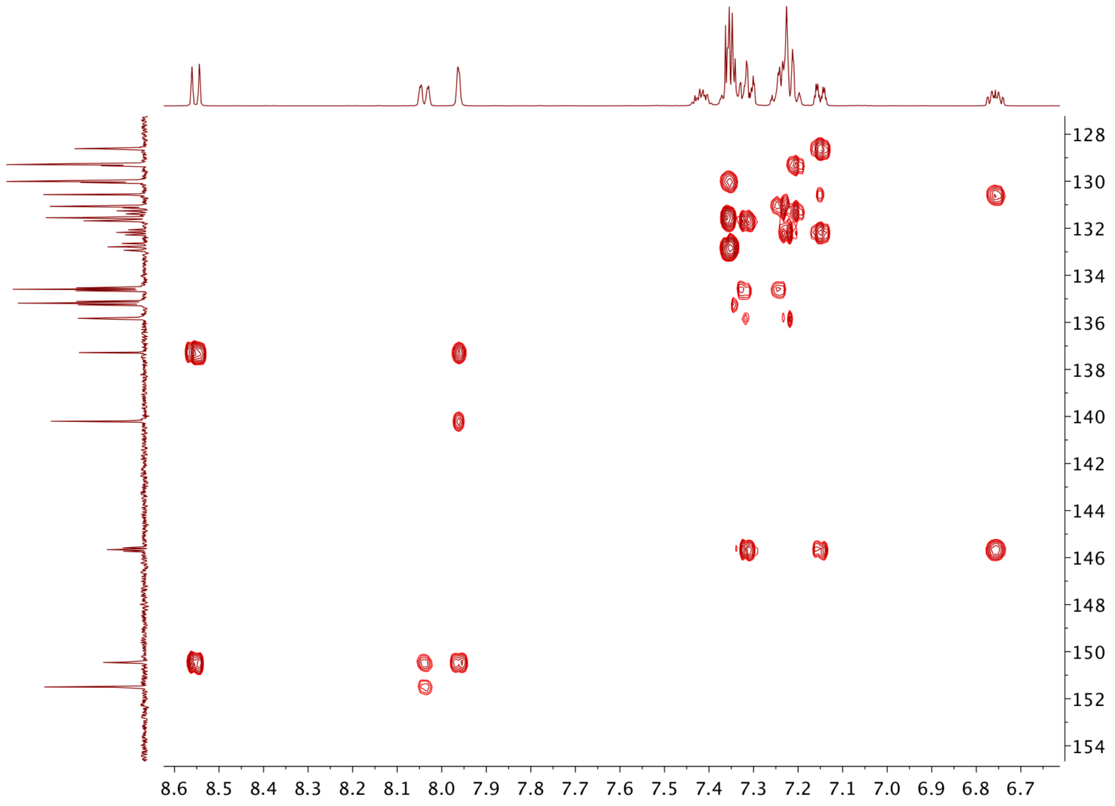

Figure S16. HMBC spectrum of [Cu(BIPHEP)(5,5'-Me_2bpy)][PF_6] (^{1}H 500 MHz, ^{13}C{^{1}H} 126 MHz, acetone-d_6, 298 K). Scales: δ / ppm.

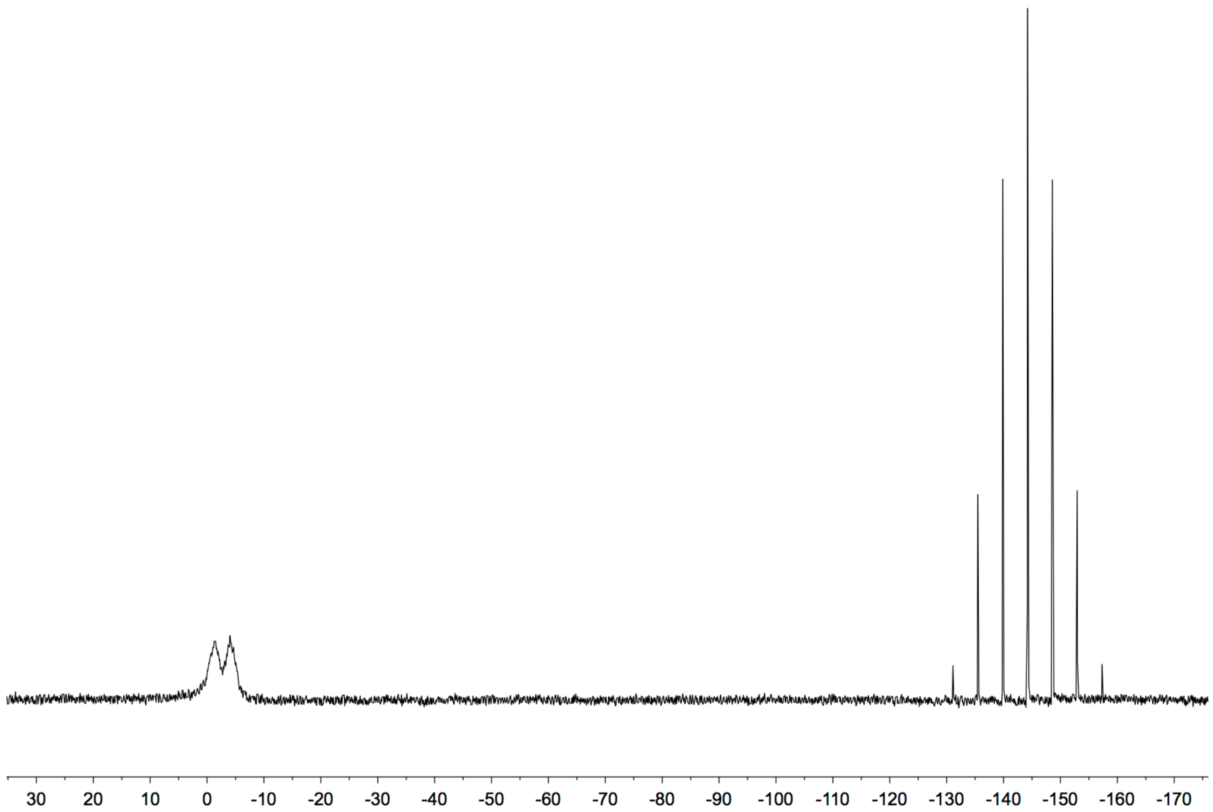

Figure S17. The $^{31}P\{^1H\}$ NMR spectrum (202 MHz, acetone-d_6, 298 K) of [Cu(BIPHEP)(6-Etbpy)][PF_6]. Scale: δ / ppm.

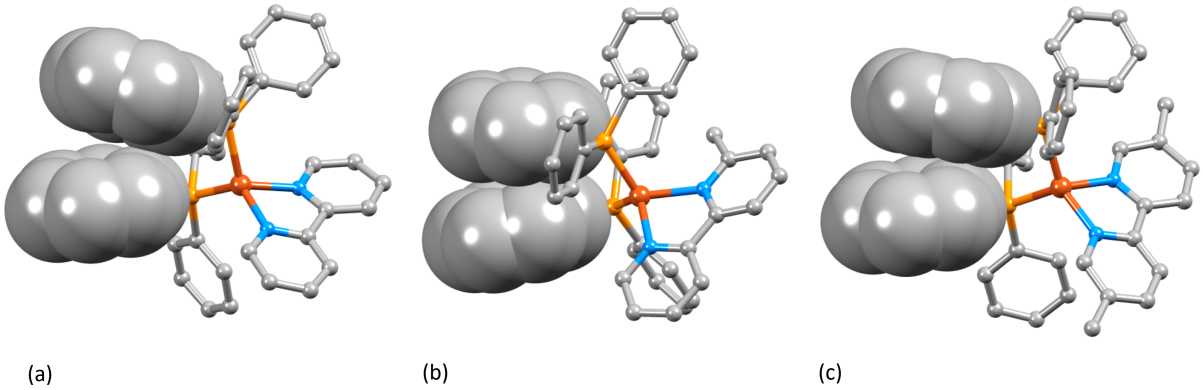

Figure S18. Space-filling representations of the π-stacking interactions between a phenyl ring of a PPh_2 group and one ring of the BIPHEP backbone in the complex cations in (a) [Cu(BIPHEP)(bpy)][PF_6]·CH_2Cl_2, (b) [Cu(BIPHEP)(6-Mebpy)][PF_6]·Et_2O·0.5H_2O and (c) [Cu(BIPHEP)(5,5'-Me_2bpy)][PF_6]·CH_2Cl_2.

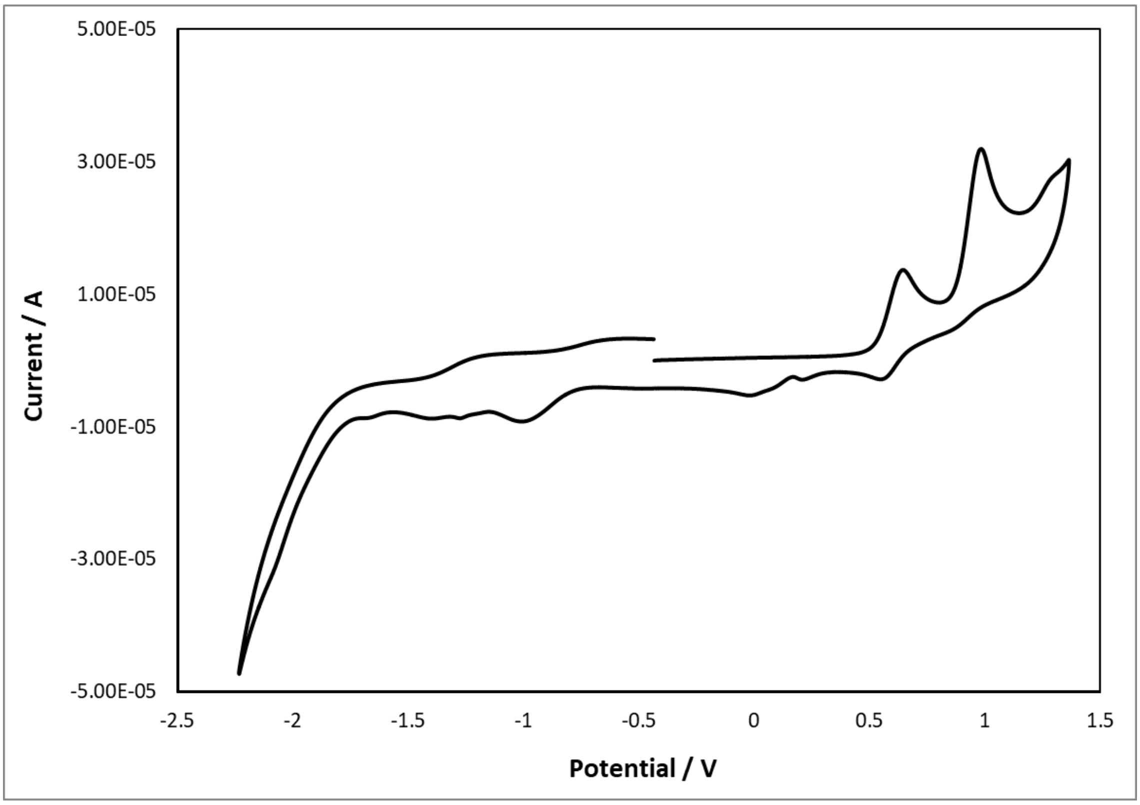

Figure S19. Cyclic voltammogram of a CH_2Cl_2 solution of [Cu(BIPHEP)(bpy)][PF_6] (vs. Fc^+/Fc, [^{n}Bu_4N][PF_6] supporting electrolyte, scan rate = 0.1 V s^{-1}).

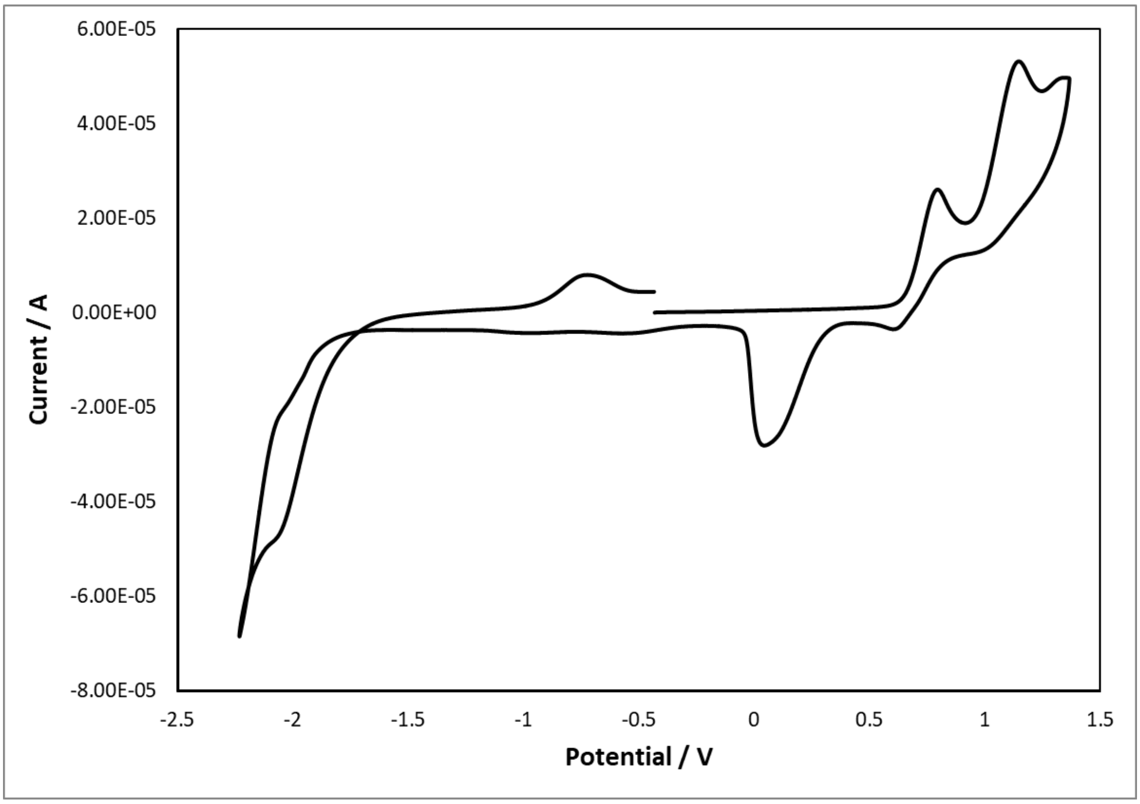

Figure S20. Cyclic voltammogram of a CH_2Cl_2 solution of [Cu(BIPHEP)(6-Mebpy)][PF_6] (vs. Fc^+/Fc, [^{n}Bu_4N][PF_6] supporting electrolyte, scan rate = 0.1 V s^{-1}).

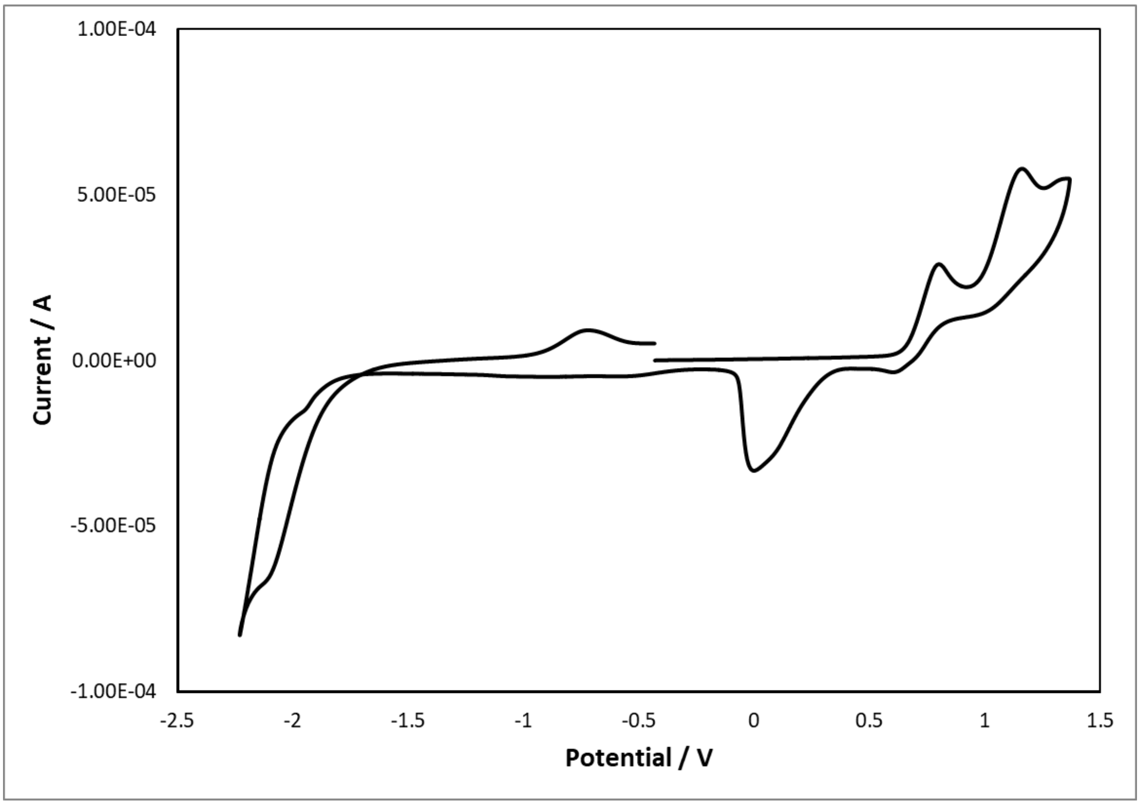

Figure S21. Cyclic voltammogram of a CH_2Cl_2 solution of [Cu(BIPHEP)(6-Etbpy)][PF_6] (vs. Fc^+/Fc, [$n$$Bu_4$N][$PF_6$] supporting electrolyte, scan rate = 0.1 V s^{-1}).

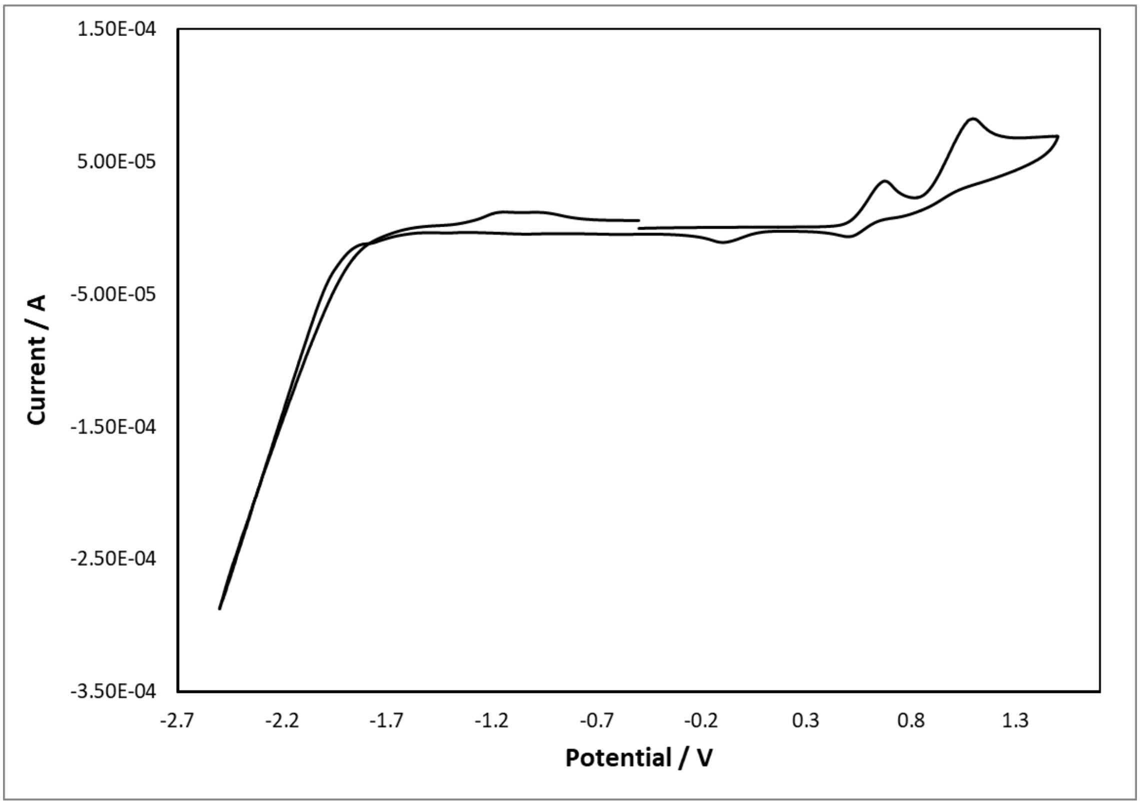

Figure S22. Cyclic voltammogram of a CH_2Cl_2 solution of [Cu(BIPHEP)(5,5'-Me_2bpy)][PF_6] (vs. Fc^+/Fc, [$n$$Bu_4$N][$PF_6$] supporting electrolyte, scan rate = 0.1 V s^{-1}).

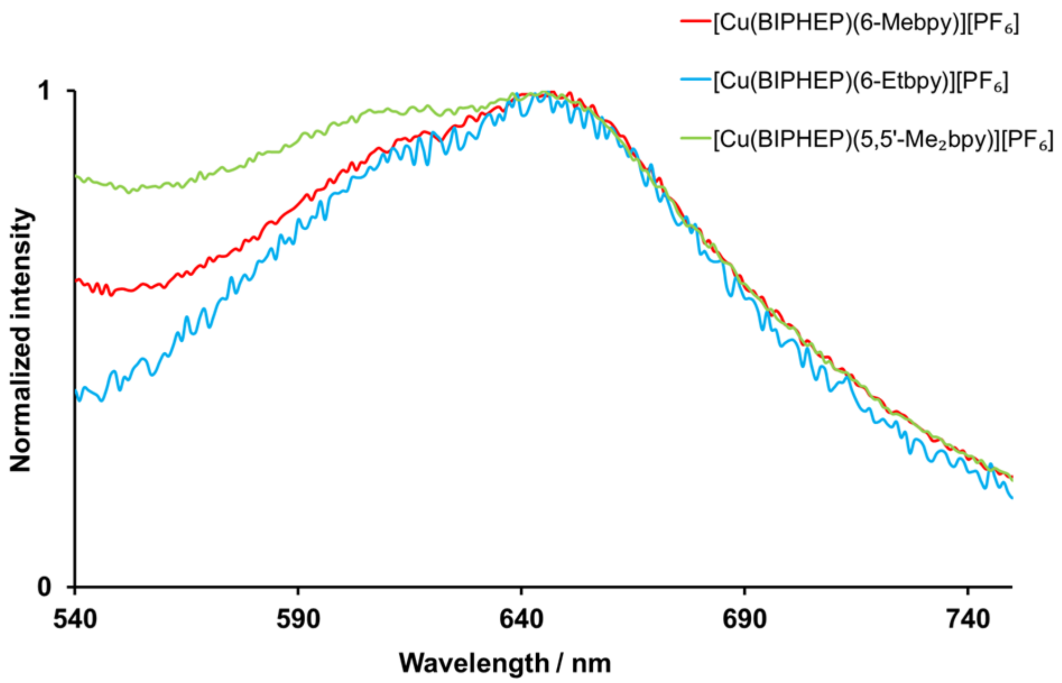

Figure 23. Normalized solution emission spectra of $[Cu(BIPHEP)(N^\wedge N)][PF_6]$ with N^N = 6-Mebpy, 6-Etbpy and 5,5'-Me_2bpy (deaerated CH_2Cl_2, 5.0×10^{-5} mol dm^{-3}). λ_{exc} = 390 nm.
